# Microgel‐Based Hierarchical Porous Hydrogel Patch with Adhesion and Resilience for Myocardial Infarction

**DOI:** 10.1002/advs.202518646

**Published:** 2026-01-05

**Authors:** Ziyang Liu, Leyan Xuan, Yingying Hou, Ting Xie, Jieting Li, Junjie Cai, Siyu Zhang, Yingling Miao, Ning Hou, Gen He, Maobin Xie, Xiyong Yu, Mingen Xu, Guosheng Tang

**Affiliations:** ^1^ Guangzhou Municipal and Guangdong Provincial Key Laboratory of Molecular Target & Clinical Pharmacology the NMPA and State Key Laboratory of Respiratory Disease School of Pharmaceutical Sciences Guangzhou Medical University Guangzhou China; ^2^ The Fourth Affiliated Hospital of Guangzhou Medical University School of Biomedical Engineering Guangzhou Medical University Guangzhou China; ^3^ School of Automation Hangzhou Dianzi University Hangzhou China

**Keywords:** 3D bioprinting, microgel, modular fabrication, myocardial infarction, porous hydrogel

## Abstract

While there has been considerable success in the 3D bioprinting of hydrogel scaffolds for tissue engineering, the application of traditional centimeter‐scale bulk hydrogels with a dense internal nanoscale network structure remains a particular challenge. Here, we present a microgel‐based modular fabrication strategy to engineer programmable hierarchically porous microgel‐based hydrogel patches (HPMPs). This strategy generates porous microgels with adjustable porosity and bio/cytocompatibility via gas‐shearing microfluidics integrated with an aqueous two‐phase system, exhibiting precise model structural fidelity, synergistic tissue adhesion, and architectural resilience. Additionally, the interconnected hierarchical porous structure of HPMP enables rapid formation of functional vascular networks in vitro. To demonstrate the broad biomedical applicability of our modular bioprinting platform, we implemented this strategy in myocardial infarction treatment. We successfully validated the application‐driven requirements via iPSC‐laden porous microgels directing cardiomyocyte differentiation and functional maturity. HPMP with Janus‐structured unilateral adhesiveness is conducive to preventing chest adhesions and cellular unilateral proliferation, migration, and angiogenesis in vivo. This microgel‐based modular fabrication strategy establishes a promising platform for targeted cardiac repair, further promoting the development of tissue engineering and regenerative medicine.

## Graphical abstract

This study develops a hierarchically porous hydrogel patch strategy (HPMP), based on gas‐shearing microfluidics and an aqueous two‐phase system to fabricate porous microgels as microgel‐based bioinks. The porous microgels with controllable porous structure exhibit excellent cellular behavior. HPMP provides a favorable platform for cellular migration and vascularisation. This strategy constructs Janus anti‐adhesion patches to promote myocardial infarction repair.

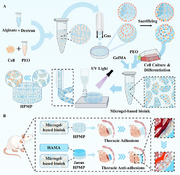

## 1 Introduction

Hydrogel scaffolds have been extensively employed in tissue engineering and regenerative medicine [1, 2]. Conventional bulk hydrogels, while valuable, are inherently constrained by nanoscale molecular crosslinking networks that restrict nutrient diffusion and cellular migration, thereby limiting regenerative efficacy. 3D bioprinting, as a cutting‐edge additive manufacturing technology, has revolutionized scaffold fabrication by enabling precise spatial control over hydrogel architectures for tissue engineering applications, thereby allowing personalized design of microporous structures to enhance cellular infiltration and tissue regeneration efficacy [3, 4, 5, 6, 7]. However, extrusion‐based bioprinting remains limited by mechanical shear stresses during printing, which compromise cell viability and functionality—a critical challenge for cell‐laden constructs.

Microgels, characterized by their microscale dimensions and high surface‐to‐volume ratios, have emerged as a promising new type of hydrogel in biomaterial research [8, 9, 10]. Cell‐laden microgels have attracted extensive attention as bioinks due to their effectively mitigate shear‐induced damage during bioprinting [11, 12]. However, conventional microgels still face significant challenges, as highly cross‐linked polymer networks restrict cellular migration and impair extracellular matrix (ECM) remodeling capacity, as exemplified by alginate‐based systems [13, 14]. Previous studies have reported porous microgels fabricated via freeze‐drying or chemical synthesis techniques [15, 16, 17, 18], but these strategies exhibit notable limitations, including the uses of the oil phase with potential cytotoxicity and biocompatibility concerns, challenges of encapsulating hydrophilic substance, cellular behavior with post‐packaging strategies, structural instability of porous networks with irregular freeze‐drying porous architectures, and compromised structural‐mechanical stability while maintaining biocompatibility. Aqueous two‐phase systems (ATPS) have emerged as a promising alternative due to their exceptionally mild, environmentally benign, and rapid spontaneous formation of porous architectures [19, 20, 21]. While ATPS‐derived porous microgels demonstrate significant advancements in mass transfer efficiency and cellular viability compared to conventional methods [22, 23, 24], the conventional microfluidic fabrication typically necessitates the introduction of an organic phase [25], which can further diminish the overall biocompatibility of the microgels and adversely impact tissue regeneration outcomes. Furthermore, although pure microgel materials possess packing characteristics and flowability, their printing precision is constrained by particle properties like size distribution, mechanical strength, secondary crosslinking capability, and the effective curing contact area between microgels. Thus, this may result in insufficient interlayer bonding strength and impose specific requirements on the microgel material.

Additionally, ischemic heart disease, as a main cardiovascular disease, remains the leading cause of death worldwide, and acute myocardial infarction (MI) is its most severe and destructive manifestation [26, 27]. Post‐MI, the cardiac microenvironment undergoes extensive remodeling characterized by irreversible cardiomyocyte loss, inflammatory activation, progressive fibrotic scarring, and inadequate vascularization [28]. While conventional pharmacological and interventional therapies provide symptomatic relief [29, 30], they fail to halt or reverse adverse ventricular remodeling. Furthermore, although stem cell‐based therapies have emerged as a promising alternative [31], they are still hampered by low cell retention, poor engraftment efficiency, and inadequate functional integration. Biomaterials such as bulk hydrogels or microgels provide structural support and microenvironmental cues critical for cardiomyocyte survival and vascularization. Yet their therapeutic impact is limited by precise control over porous architecture for nutrient diffusion and cell migration, insufficient niche for functional vasculature development, mismatched mechanical properties, and dynamic adaptability of the cardiac tissue [32, 33]. Therefore, there is a critical need to develop multifunctional biological repair scaffolds capable of enhancing cell migration, promoting angiogenesis, and supporting dynamic tissue remodeling. However, current hydrogel systems struggle to simultaneously fulfil these multimodal demands.

**FIGURE 1:**
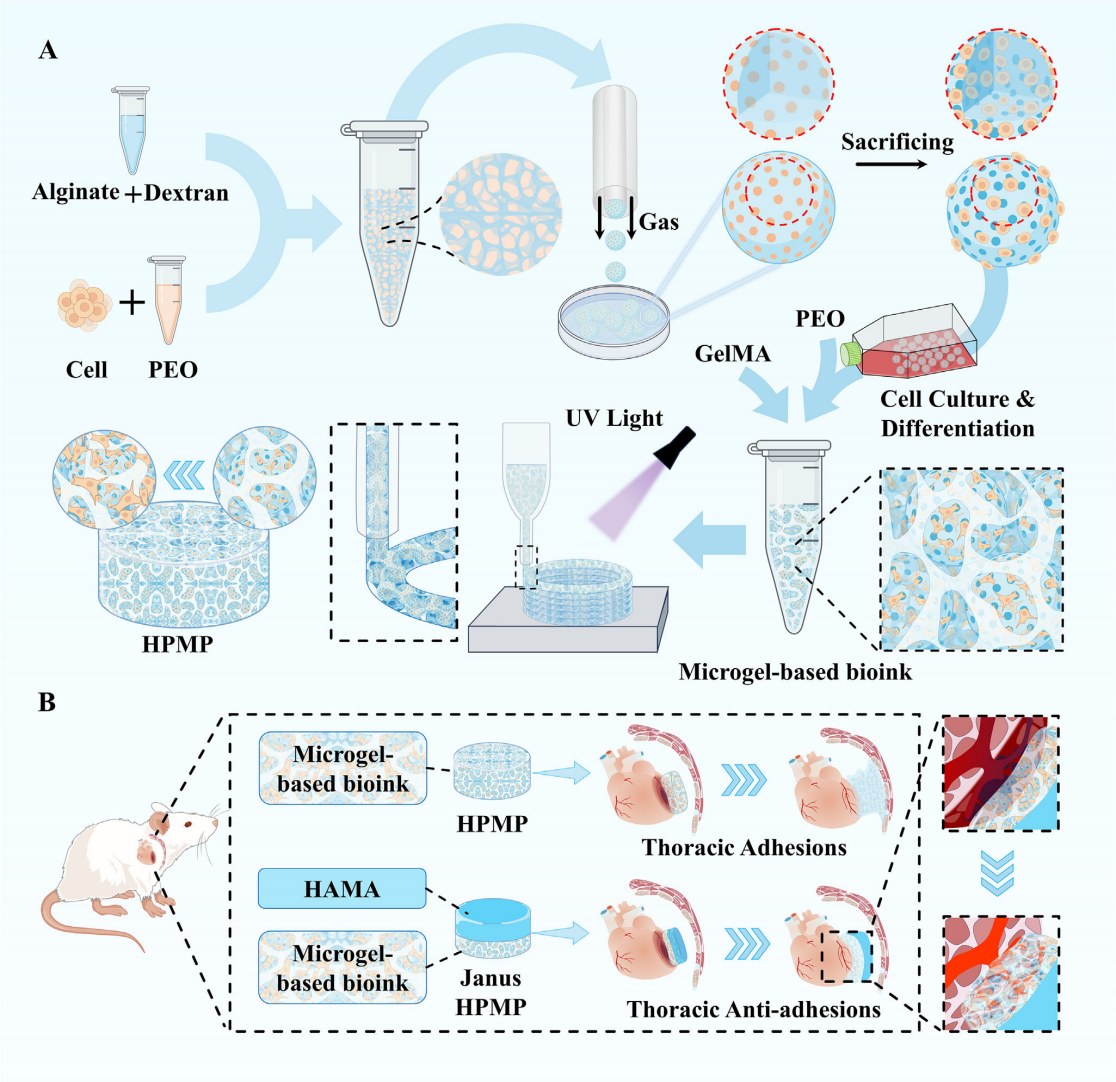
Schematics of the modular fabrication strategy for tissue regeneration. (A) Fabrication of porous microgels via gas‐shearing microfluidics and subsequent 3D bioprinting of microgel‐based hierarchically porous constructs; (B) Application of HPMP strategy for MI repair.

Herein, to address the multifaceted requirements for tissue regeneration and repair, we present a modular manufacturing methodology for engineering hierarchical porous hydrogel patches (HPMP) through a two‐stage microgel‐based fabrication process (Figure 1A). We demonstrated that our strategy was mainly based on gelatin methacrylate (GelMA), as the continuous phase of bioink, and poly (ethylene oxide) (PEO), as the sacrificial phase of bioink, mixed with alginate (Alg) porous microgels. GelMA, a photopolymerizable hydrogel derived from gelatin, has been extensively employed in the fabrication of cell‐laden 3D tissue constructs, a utility attributed to its controllable photocross‐linking behavior, biocompatibility, biodegradability, inherent bioactive properties, and tunable physical characteristics. PEO, a biocompatible polymer with exceptional hydrophilicity mediated by hydrogen‐bonding interactions, has emerged widely in biomedical engineering, enabling applications ranging from controlled drug delivery systems to bioengineered tissue scaffolds. Alg, an anionic polysaccharide derived from brown algae, is utilized in biomedical applications ranging from drug delivery to tissue engineering, owing to exceptional biocompatibility and dynamic gelation properties mediated by divalent cations. First, in our strategy, we initially present a robust method to address the challenge of fabricating microporous Alg microgels by combining an ATPS with gas‐shearing microfluidics. The ATPS is composed of Alg mixed with dextran (Alg‐Dex) and PEO, among which Alg‐Dex is the continuous phase, and PEO is the sacrificial phase. Subsequently, these microgels function as structural units for 3D printing. We employed main bioinks with GelMA as the continuous phase and PEO as the sacrificial phase, combined with porous microgels, facilitating the controlled assembly of HPMP with hierarchically porous structures. The microgel‐based bioink exhibited robust printing capabilities. The HPMP exhibited mechanical properties with adhesion and resilience, and favorable cellular behavior, enabling supporting cell migration and vascularization. Second, to demonstrate the potential of our strategy to flexibly address major global health challenges, we utilized the HPMP strategy to confront the significant issue of cardiovascular disease by focusing on the repair of myocardial infarction (Figure 1B). We employed porous microgels loaded with induced pluripotent stem cells (iPSCs) to induce cardiomyocyte differentiation. In the context of MI repair, to address post‐transplantation‐generated thoracic adhesions, we have developed an anti‐adhesion patch‐based HPMP strategy via hyaluronic acid methacrylate (HAMA). HAMA, a photosensitive derivative of hyaluronic acid, characterized by exceptional biocompatibility, high water retention, and densely crosslinked architectures with lubricating mechanical properties, has wide applications in tissue engineering and regenerative medicine. We utilized microgel bioink and HAMA bioink for printing a Janus structure with anti‐adhesion properties. This optimization of this methodology further enhances myocardial infarction repair and demonstrates the flexibility of our strategy for on‐demand adjustment. Overall, we propose a microgel modular strategy to establish a versatile bioprinting platform. This strategy demonstrated integration of cell differentiation, mechanical support, and tissue repair, thereby demonstrating significant potential for advancing regenerative therapies.

## 2 Results

### 2.1 Fabrication of Porous Microgels via Aqueous Two‐Phase System

**FIGURE 2:**
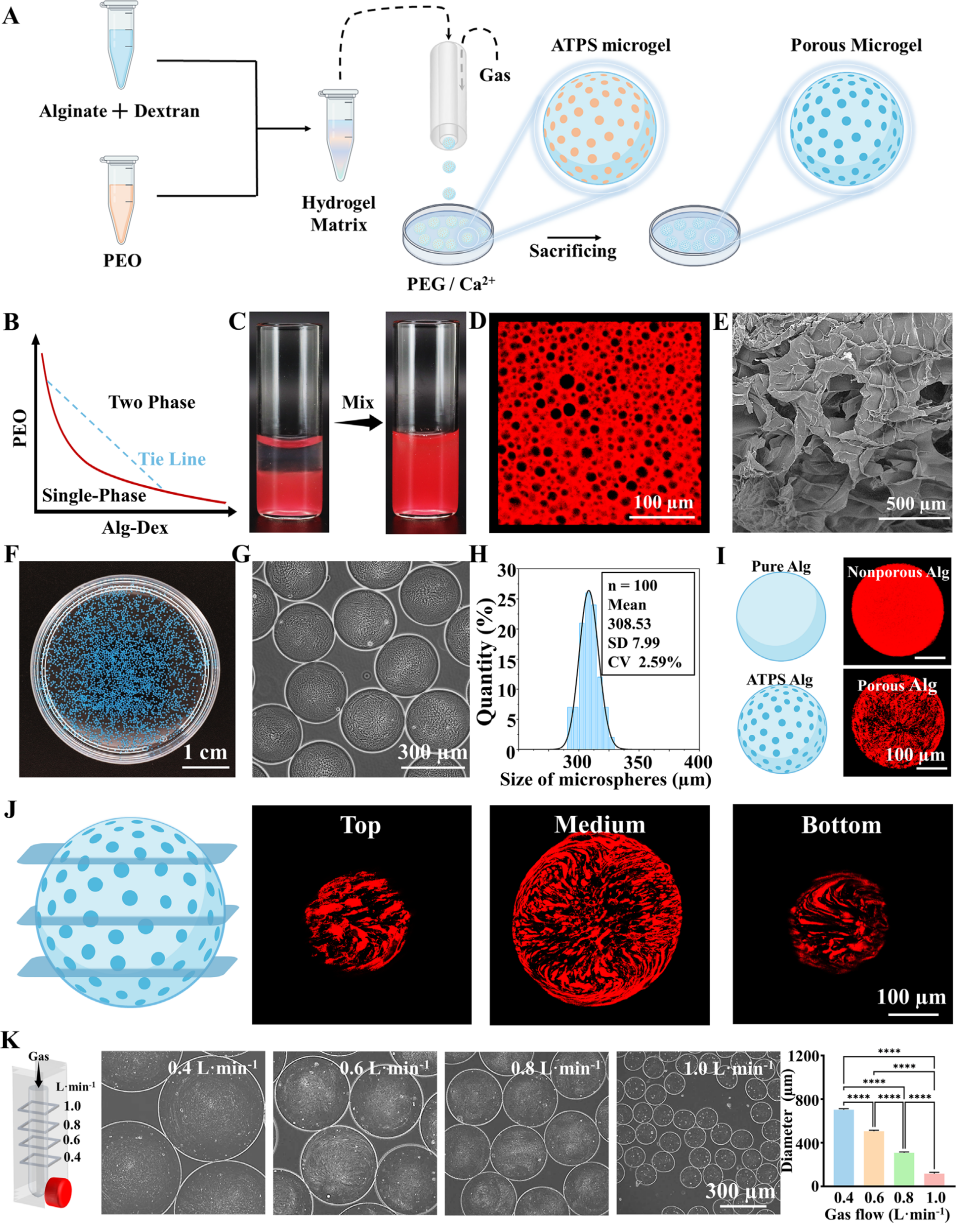
Fabrication of Aqueous Two‐Phase Microporous Microgels via Gas‐Shear Microfluidics. (A) Schematic diagram of the fabrication of porous microgels via gas‐shearing with an aqueous two‐phase system. (B) The phase diagram of the Alg‐Dex mixed aqueous phase and the PEO aqueous phase. (C) Photographs of Alg‐Dex (red) and PEO before and after vortexing. (D) Fluorescence images of the precursor solutions of the Alg‐Dex (red) and PEO. (E) SEM images of the Alg‐Dex and PEO aqueous two‐phase hydrogels after Ca^2+^ curing and washed. (F) Photograph of the porous microspheres via gas‐shearing microfluidic; (G) The micrograph of the porous microspheres; (H) Size distribution profile of the porous microspheres; (I) The fluorescence image and 3D fluorescence imaging of pure Alg non‐porous microspheres and Alg porous microspheres; (J) The schematic diagram and fluorescence images of Alg porous microsphere sections; (K) Schematic diagram and phase contrast micrographs showing the fabrication of porous microspheres under varying gas flow rates, n = 100; one‐way ANOVA; ^*^ *P* < 0.05, ^**^ *P* < 0.01, ^***^ *P* < 0.001, ^****^ *P* < 0.0001. Data are presented as mean values ± SDs.

To construct porous microgels that promote cell growth and migration, we combined a novel gas‐shearing microfluidic strategy and ATPS, generating microgels with highly interconnected micropores (Figure 2A). Interconnected porosity was achieved by formulating a cell‐laden ATPS bioink through room‐temperature mixing of an Alg‐Dex pre‐gel solution with PEO at an optimized volume ratio. Stable biphasic stratification confirmed successful ATPS formation, directly enabling one‐step bioink preparation (Figure 2B,C; Figure S1A,B). To validate the phase separation in this bioink, 0.2% red fluorescent nanoparticles were mixed into the Alg‐Dex phase for confocal imaging. As shown in Figures 2D and S1C, PEO can form discrete droplets in the Alg‐Dex solution and porous network after crosslinking. Phase‐specific dissolution of PEO droplets and selective leaching of Dex result in stabilized microporous networks. The scanning electron microscopy (SEM) image further confirmed the microporous structure (Figure 2E).

Using this ATPS bioink, we established a gas‐shearing microfluidic platform for fabricating porous microgels (Figure S2A) [34]. The system integrates a digital injection pump, a coaxial nozzle, and a collecting bath. ATPS precursor solutions underwent continuous gas‐shearing processing. Precisely regulated nitrogen flow through the coaxial nozzle's annular gap exerted shear forces overcoming liquid surface tension to produce monodisperse droplets continuously (Figure S2C, D). Using a 2% calcium ion (Ca^2^^+^) solution as the collecting bath, non‐spherical porous microgels were observed. It may be attributed to interfacial instabilities arising from the rapid solidification of Alg and the delayed dissolution of the PEO sacrificial phase. To address this, we introduced polyethylene glycol (PEG) as a stabilizing agent into the collecting bath. Systematic concentration‐gradient optimization of the Ca^2^^+^/PEG system revealed that 1% PEG enabled spherical microgel formation (Figure 2F), while other concentrations produced special morphologies of microgels (Figure S3), establishing PEG as a critical regulator of crosslinking kinetics.

As shown in Figure 2G,H, porous microgels with high monodispersity can be fabricated. To confirm successful porous structure formation, we characterized the internal morphology of the microgels. The microgels exhibited a well‐defined porous architecture, in striking contrast to the non‐porous structure of pure Alg microgels (Figure 2I; Figure S4). The porous microgels exhibited excellent 3D structural continuity, with uniformly interconnected channels persisting throughout all cross‐sections of the cross‐linked matrix, confirming a fully integrated porous framework (Figure 2J; Figure S5A). To further confirm interconnected porosity, we mixed Alg‐Dex and PEO with fluorescent nanoparticles, respectively, observing the pore space of PEO and then dissolution over time (Figure S5B). The diameter of porous microgels can be precisely tuned from 100 to 700 µm by adjusting key processing parameters, including nozzle diameter and gas flow velocity. Systematic optimization of these variables enables robust and reproducible fabrication of size‐controlled microgels (Figure 2K).

**FIGURE 3:**
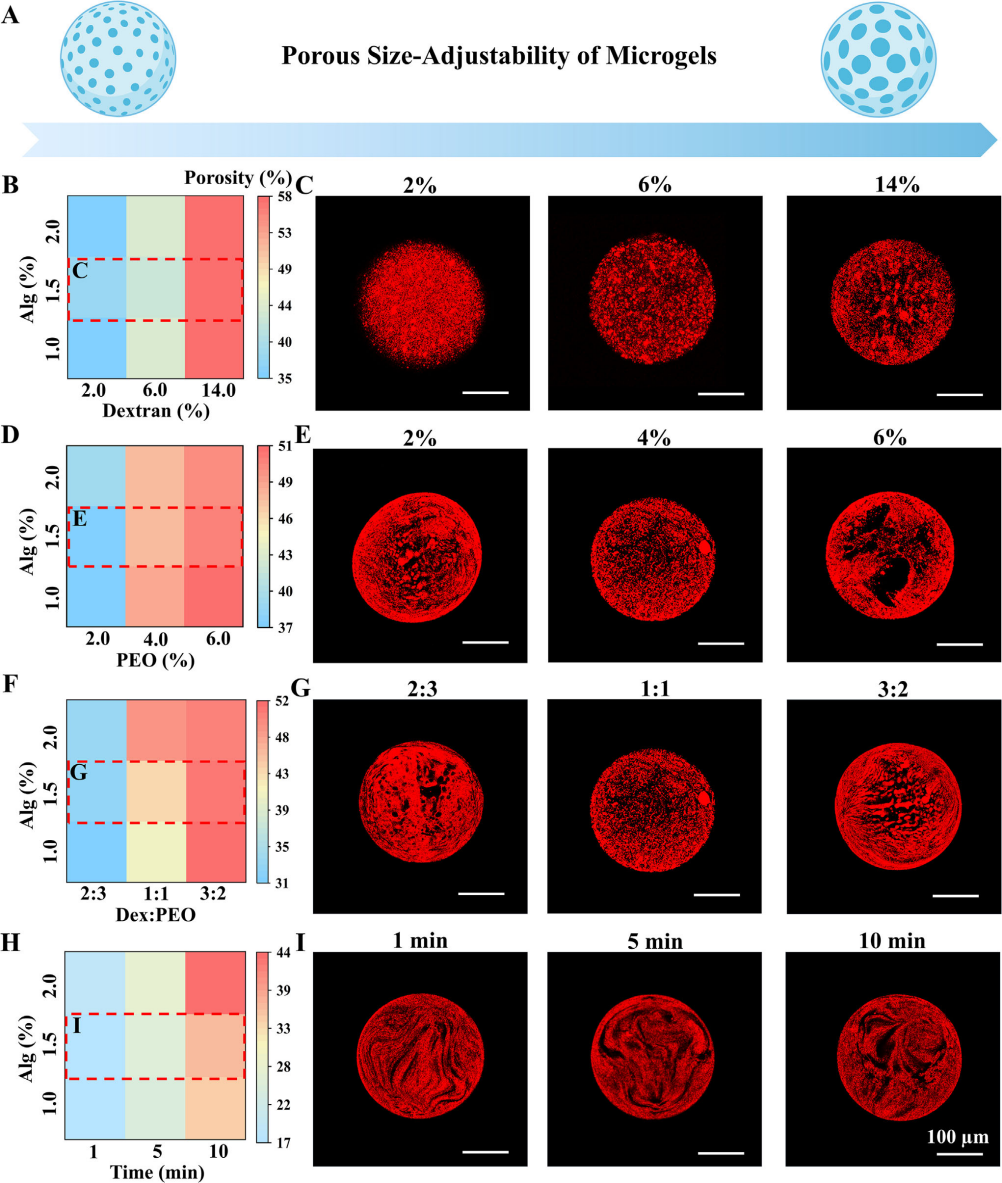
Robust Size‐adjustability Porosity of Microporous Microgels via Modulations of ATPS. (A) Schematic of controllable pore size adjustment in porous microgels; (B) Heat map of porosity regulation via Dex concentration variation in aqueous two‐phase porous microgels; (C) Fluorescence image of ATPS porous microgels with Dex concentration adjustment; (D) Heat map of porosity control by PEO concentration variation in aqueous two‐phase porous microgels; (E) Fluorescence image of ATPS porous microgels with PEO concentration modification; (F) Heat map of porosity regulation through mixing ratio variation in aqueous two‐phase porous microgels; (G) Fluorescence image of ATPS porous microgels with mixing ratio adjustment; (H) Heat map of porosity control by standing time variation in aqueous two‐phase porous microgels; (I) Fluorescence image of ATPS porous microgels with standing time modification.

Based on the adjustability of the ATPS, porous Alg microgels with precisely tunable porosity can be fabricated (Figure 3A). Systematic modulation of phase concentrations, component ratios, and gelation duration enabled precise pore size and porosity control without post‐processing. When increasing dextran concentration (continuous phase, 2% PEO fixed), porosity rose proportionally (Figure 3B,C). Similarly, higher PEO concentrations in Alg‐Dex mixtures enhanced porosity (Figure 3D,E). The effect of phase ratio (continuous phase: sacrificial phase) on porosity modulation was also investigated. Volumetric phase ratio optimization revealed that maximal porosity was yielded at 3:2, with other ratios reducing pore formation (Figure 3F,G). Given the phenomenon of complete phase separation of the ATPS bioink over time, extended ATPS standing duration at fixed concentration and ratio further augmented porosity (Figure 3H,I). In general, these ATPS parameters governed microgel porosity from 17 to 60% (Figures S6 and S7), providing foundational insights for engineering porous microgels in cell culture applications.

### 2.2 Cellular Behaviors of Porous Microgels

**FIGURE 4:**
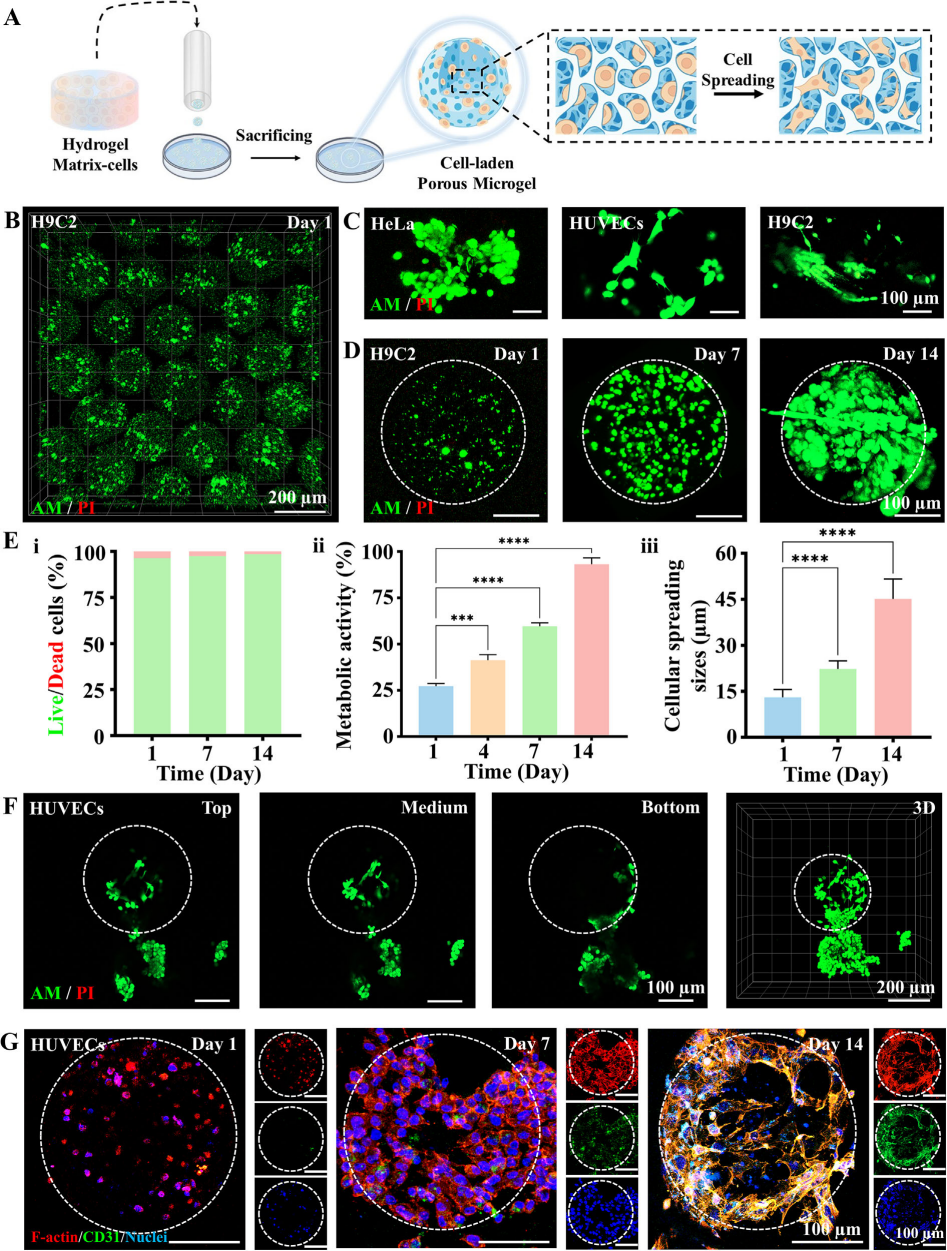
Cellular Behaviors on Microporous Microgels via Aqueous Two‐Phase. (A) Schematic illustration of cell‐laden porous microgel culture; (B) Fluorescence 3D micrograph of Live/Dead staining for H9C2‐laden porous microgels at 1 day culture; (C) Fluorescence images of Live/Dead staining for three cell types (HeLa, HUVECs, and H9C2) respectively encapsulated in porous microgels at 7 day culture; (D) Fluorescence images of Live/Dead staining for H9C2‐laden porous microgels at 1, 4, and 7 day culture; (E) Quantification results of cell viabilities, metabolic activities, and cellular spreading sizes, n = 3; one‐way ANOVA; ^*^ *P* < 0.05, ^**^ *P* < 0.01, ^***^ *P* < 0.001, ^****^ *P* < 0.0001. Data are presented as mean values ± SDs. (F) Fluorescence microscopy images of Live/Dead staining for HUVECs‐laden porous microgels at 1, 4, 7 days of culture; (G) Fluorescence images of the HUVECs‐laden porous microgels over 14 days of culture stained for F‐actin (red), CD31 (green), and nuclei (blue).

As the oil‐free processing of gas‐shearing microfluidics and ATPS that preserves high cell viability [35, 36], we directly encapsulated cells in bioink to fabricate porous microgels (Figure 4A). Cells were distributed homogeneously with superior cellular viability at the first 24 h (Figure 4B). To further evaluate the cytocompatibility of ATPS porous microgels, we respectively encapsulated human cervical carcinoma cells (HeLa), human umbilical vein endothelial cells (HUVECs), and rat cardiomyocytes (H9C2) into porous microgels using the above methodology. All cells demonstrated high viability after 7 days, with progressively spreading and adopting spindle‐shaped morphologies throughout the culture period (Figure 4C). Compared with cell‐laden non‐porous 1.5% Alg microgels, the cell‐laden porous microgels maintained higher cellular viability after 14 days (Figures S8 and S9). To further confirm cellular growth within the porous microgels, H9C2‐encapsulated porous microgels were observed over 14 days using by cellular viewer. As shown in the Figure S10, H9C2 in microgel exhibits robust proliferation and migration capabilities, enabling growth throughout the microgels. To demonstrate cellular proliferation, we assayed cell viability and metabolic activity characterized by Live/Dead staining, CFSE, and PrestoBlue assays (Figure 4D,E; Figure S11A). The H9C2 cells gradually spread and migrated from the microgels while retaining strong metabolic activities with increasing cellular spreading areas (Figure 4Eii,iii), and the HUVEC‐laden microgels demonstrated pronounced cell proliferation efficacy (Figure S11B).

Precise modulation of ATPS parameters enabled reproducible fabrication of uniform porous microgels with tunable porosity as demonstrated above. To identify the effect of microgel porosity on cell behavior, H9C2 cells were cultured in different porosities for 7 days. Cells in porous microgels with porosity from 17% to 58% maintained high viability (Figure S12). In addition, to evaluate the impact of microgel morphological variations on cellular behavior, we engineered two distinct microgel types, including ellipse‐shaped and drop‐shaped. We observed that morphological variations showed no significant impact on proliferation or viability (Figure S13).

Given the critical role of cellular migration in tissue regeneration and repair processes, we assessed migratory ability and functional expression in HUVEC‐laden porous microgels over 28 days. HUVECs in the microgels exhibited outward migration within 14 days, culminating in complete microgel detachment with sustained proliferation by 28 days (Figure 4F; Figure S9). F‐actin staining revealed progressive cellular growth and reorganization from 1 day, eventually forming into densely packed cellular aggregates spanning within microgels by 14 days. Furthermore, CD31 expression intensified and co‐localized with migrating cells, confirming sustained functional bioactivity (Figure 4G; Figure S14). HeLa‐laden porous microgels fully migrated out and adhered to the culture dish within 7 days, further confirming the porous microgels as a robust platform for cellular proliferation and migration (Figure S15). These results illustrated that ATPS porous microgels provide excellent cytocompatible microenvironments that preserve cellular functionality while enabling complex migratory behaviors.

### 2.3 Printability of Microgel‐Based Bioink

**FIGURE 5:**
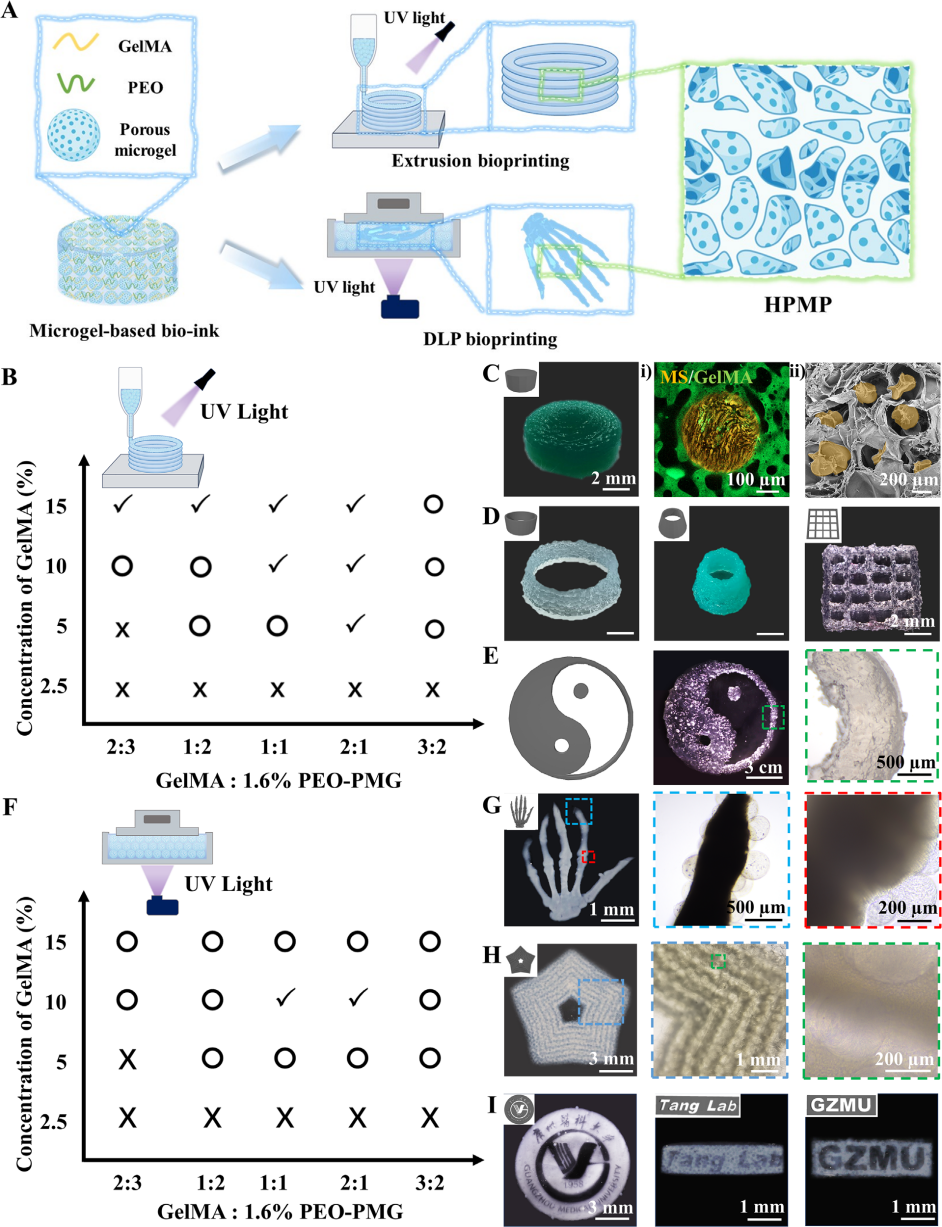
Fabrication of Hierarchically Porous Hydrogel via Microgel‐based 3D Bioprinting. (A) Schematic illustration of microgel‐based hierarchically porous hydrogel; (B) Printability map of microgels‐based bioink via extrusion printing (✓: well‐definite printable, ○: printable but the shape was not agreement with CAD, ×: less printable); (C) CAD and photographs of HPMP with fluorescence micrographs (i) and SEM (ii); (D) CAD and photographs of hollow cylinder constructs, narrow‐hollow cylinder constructs, grid lattice constructs; (E) CAD and microscopic images of Tai Chi constructs; (F) Printability map of microgels‐based bioink via DLP printing (✓: well‐definite printable, ○: printable but the shape was not agreement with CAD, ×: less printable); (G) CAD and photographs of hand bone and microscopic images; (H) CAD and photographs of pentagonal stars with microscopic images; (I) CAD and photographs of Guangzhou Medical University, “GZMU” and “Tang Lab” emblem.

Although 3D bioprinting of cell‐laden scaffolds holds broad regenerative potential, achieving physiological functionality requires tissue‐mimetic cellular aggregates rather than dispersed cell populations [37, 38]. Building on our demonstration that porous microgels maintain high cellular vitality and enhance cellular migration, we engineered a novel hierarchical porous hydrogel bioink with porous microgels to optimize hierarchical structure and cell interactions. Our previous work demonstrated that the ATPS of GelMA and PEO enable facile bioprinting of micropore‐forming hydrogel [19]. We hypothesized that this system, with further optimization of the GelMA‐PEO system with porous microgels (GelMA‐PEO‐PMG), could enable direct microgel‐based 3D bioprinting (Figure 5A). To validate this hypothesis, we first conducted rheological characterization of the microgel‐based bioink (Figure S16). In frequency sweep testing, the bioink exhibits performance characteristics comparable to those of pure GelMA (Figure S16A). Within the low‐frequency range, the loss modulus (G′″) surpasses the storage modulus (G′), indicating a higher degree of fluidity, whereas in the high‐frequency domain (G′ > G″’), the bioink reflects enhanced gel‐like behavior. This may suggest the microgel‐based bioink with potential applicability in the bioprinting process. During temperature sweep tests, the microgel‐based bioink demonstrates thermosensitive gelation, with an inflection point occurring about 20°C (Figure S16B). In low temperature (G′ > G″), microgel‐based bioink with robust gel characteristics may contribute to structural integrity during printing by mitigating collapse. Furthermore, assessment of the bioink's viscosity revealed a gradual decrease from 0℃ to 40°C, stabilizing into a plateau phase between 30℃–35°C (Figure S16C). In contrast to the continuous decline observed in pure GelMA, the relative viscosity stability of the bio‐ink implies a capacity to maintain flowability during extrusion while facilitating structural rigidity post‐printing. Accordingly, we further assessed GelMA‐PEO‐PMG bioink printability to validate this hypothesis.

We applied this bioink to the extrusion‐based bioprinting, the most widely used modality of 3D bioprinting for additive manufacturing. Extrusion‐based bioprinting requires bioinks exhibiting both liquid‐phase flowability and solid‐like gelation to enable the construction of 3D architectures via continuous nozzle extrusion [39, 40]. Using the thermoresponsive properties of GelMA, which facilitate reversible physical crosslinking prior to photo‐crosslinking and increase the viscosity within specific temperature ranges, we utilized pre‐cooled GelMA‐PEG‐PMG bioinks and low temperature to maintain stable extrusion. To evaluate the extrusion printing behavior of microgel‐based bioinks, we characterized bioink extruding morphology of filament with parameterized gradients of GelMA concentration (2.5%–15%) and GelMA: PEO‐PMG ratios (2:3‐3:2). As experimentally illustrated in Figure S17A, GelMA concentrations over 5% facilitated enhanced physical crosslinking, enabling continuous, stable extrusion and elongated filament formation. While low GelMA concentrations (<5%) showed insufficient gelation, resulting in discontinuous droplet extrusion. Conversely, elevated PEO content increased the aggregation of microgels at the nozzle, inducing nozzle‐entrance coiling and intermittent flow obstruction followed by pressure‐driven burst extrusion without filament formation. While above 10% GelMA, the bioink exhibited robust printability with uniform filament formation under most blend ratios, except when GelMA: PEO‐PMG rate exceeded 2:3 induced microgel aggregation, leading to being stuck in the nozzle head. The pattern printing experiment also confirmed the above conclusion (Figure S17B). These findings indicate that simultaneous optimization of GelMA concentration (≥10%) and PEO‐GelMA phase ratio is critical to balance curing kinetics and flow properties, thereby developing a novel bioink with enhanced printability and structural fidelity (Figure 5B). Using optimized GelMA‐PEO‐PMG bioink enabled direct printing of several representative 3D constructs, including cylindrical constructs, hollow cylinder constructs, narrow‐hollow cylinder constructs, grid lattice constructs, and Tai Chi constructs (Figure 5C–E). To illustrate the hierarchical porous structure in the printed model, we characterized the interior structure by microgels conjugated with red fluorescent nanoparticles and the GelMA matrix with green. The printing architecture exhibited a distinct hierarchical porous structure after photocross‐linking (Figure 5C,i). This is also further confirmed by SEM (Figure 5C,ii), demonstrating stable retention of the hierarchical porosity.

Expanding the versatile printability of the microgel‐based bioink, we employed digital light processing (DLP) printing to fabricate complex structures with designable features. As a stereolithographic technique, DLP bioprinting utilizes dynamic light projection to photopolymerize photosensitive bioinks, enabling high‐precision layer‐by‐layer fabrication [41, 42]. Thus, DLP bioinks require photoresponsiveness with high transparency for penetration depths of light and liquid fidelity with low viscosity for printing platform lifting [43]. We systematically assessed printability by analyzing deviations between printed constructs and their computer‐aided design (CAD) models. GelMA‐PEO‐PMG solutions fabricated hand bone models within a thermostatically controlled printing platform via DLP printing platform (Figure S18). Similar to extrusion‐based bioprinting, 2.5% GelMA failed to support viable printing due to inadequate photocross‐linking for effective gelation. While reducing the PEO‐PMG phase proportion partially restored structural integrity, 5% GelMA formulation still exhibited persistent fragmentation despite enhanced curing. Optimal performance was achieved at 10% GelMA, where all tested phase ratios enabled basically hand bone formation, with GelMA: PEO‐PMG ratios of 1:1 and 2:1 exhibiting the highest morphological fidelity to CAD models. Increasing GelMA concentrations to 15% induced excessive crosslinking in all ratios, particularly the 3:2 GelMA: PEO‐PMG formulation, causing complete structural fusion. Printability map in Figure 5F indicated the printable areas, conclusively identifying 10% GelMA with PEO‐PMG ratios of 1:1 or 2:1 as optimal for DLP bioprinting. Utilizing this optimized formulation and printing parameters, we fabricated representative 3D constructs featuring complex internal and external architectures. Notably, the successful printing of hand bone models with a distinct porous structure verified the hierarchical porosity within the DLP printed constructs (Figure 5G). Additionally, hollow pentagonal stars with well‐defined matryoshka‐like constructs were successfully printed, validating the bioink's capability for generating intricate 3D porous scaffolds (Figure 5H; Figure S19). To further investigate the printability of complex models by GelMA‐PEO‐PMG bioink, we respectively employed CAD of the Guangzhou Medical University emblem, “GZMU” and “Tang Lab” abbreviations with comprising an annular frame with hollow bilingual (Chinese‐English) letters. These structures revealed printing models with interwoven internal structures, reduced‐scale constructs, and clear edges (Figure 5I). The fabrication with architecturally complex features, including nested architecture, biological structure, and hollow edges, demonstrates the printability of GelMA‐PEO‐PMG bioink, while highlighting a microgel‐based strategy with potential for fabricating patient‐specific tissue scaffolds customized for structural restoration. The hierarchical porous architecture within the printing models further provides a critical cellular microenvironment platform essential for regenerative tissue engineering applications. To elucidate the cellular behavior of the printing constructs, we post‐printed cultured cell‐laden microgel bioinks and subsequently evaluated cell morphology and viability via live/dead assays and immunofluorescence staining. As shown in the Figure S20, the printed scaffold demonstrated excellent cellular compatibility after seven days of culture, exhibiting favorable spreading behavior within the biomaterial matrix.

### 2.4 Adhesion and Resilience of the Porous Microgel and HPMP

**FIGURE 6:**
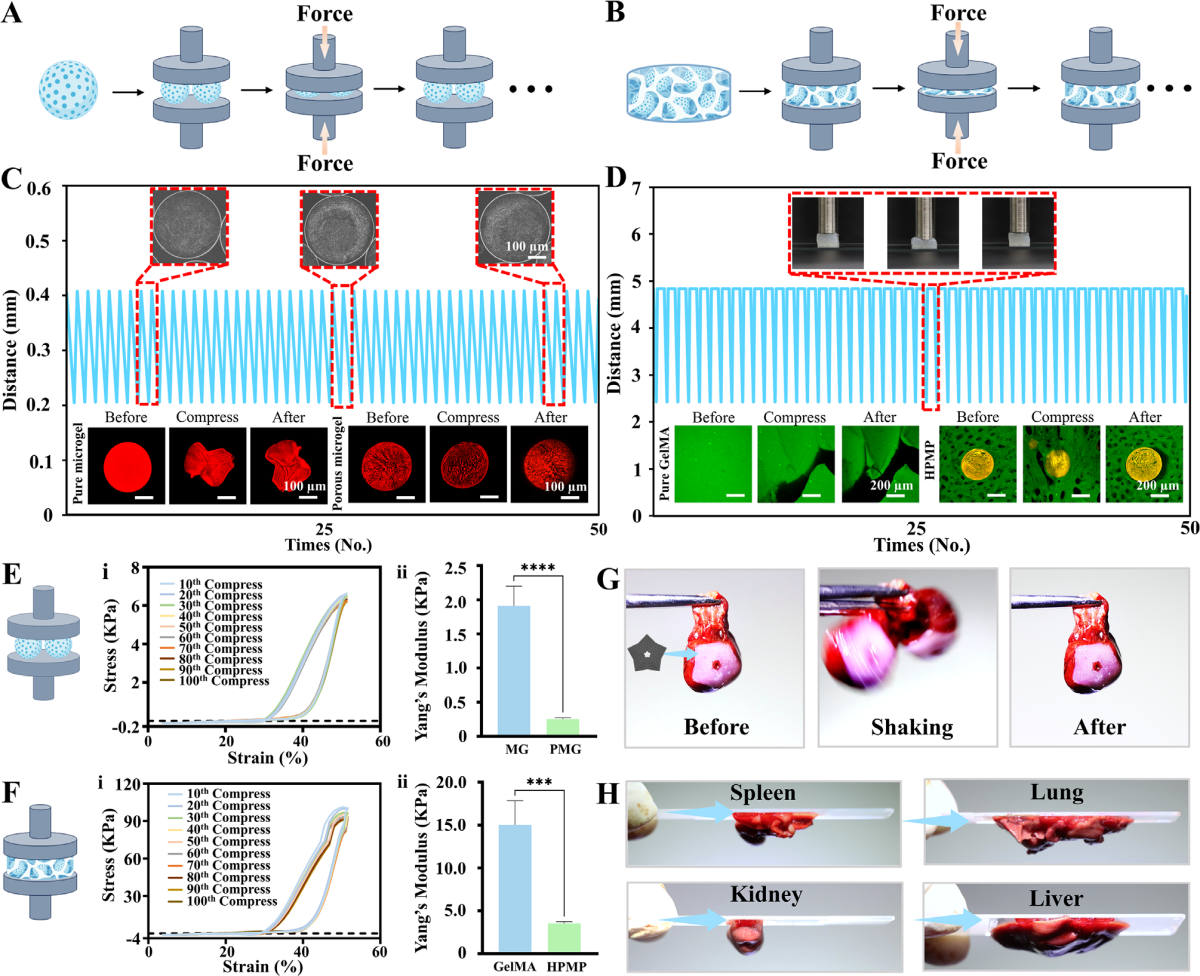
Mechanical Characterization of Porous Microgels and Hierarchically Porous Hydrogel Constructs. (A, B) Schematic of compression test procedure of porous microgels and HPMP; (C) The curve of time‐displacement and morphologies and fluorescence micrographs of porous microgels and pure microgels with 50 cycles of compression test; (D) The curve of time‐displacement inserting photographs of HPMP in compression test process and fluorescence micrographs of HPMP and pure GelMA patch with 50 cycles of compression test; (E) The cycles compression curve and yang's modulus of porous microgels; (F) The cycles compression curve and yang's modulus of HPMP, n = 10; t text; ^*^ *P* < 0.05, ^**^ *P* < 0.01, ^***^ *P* < 0.001, ^****^ *P* < 0.0001. Data are presented as mean values ± SDs; (G) Photographs of HPMP adhered to the heart; (H) Photographs of HPMP‐adhesion with multiple organs (liver, spleen, lung, kidney) onto the glass after 6 h.

The mechanical properties of biofabricated constructs critically influence tissue engineering applications, particularly resilience to accommodate dynamic tissue contraction and adhesive characteristics to promote tissue regeneration [44, 45]. To experimentally evaluate these properties, we performed cyclic compression testing (1 N load, 50% strain, room temperature) using an SMS Texture Analyzer to characterize porous microgels and HPMP (Figure 6A,B).

Porous microgels exhibited high reversible responsiveness and superior compressive recovery under cyclic loading compared to non‐porous alginate controls, maintaining dimensional parameters and pore architecture comparable to their original relaxed state (Figure 6C). Following 100 compression cycles, porous microgels displayed well‐shaped compression profiles with 87.5% retention of maximum stress (Figure 6E i) and demonstrated significantly reduced Young's modulus relative to non‐porous counterparts (Figure 6E ii). This enhanced resilience originates from stress‐accommodating structural deformation within the microporous architecture, where microporous structural collapse dissipates mechanical energy without fracture, enabling near‐complete shape recovery upon load removal.

Concurrently, cyclic compression testing demonstrated HPMP exhibiting highly reversible elastic recovery, while pure GelMA hydrogels underwent irreversible deformation (Figure 6D). HPMP constructs maintained structural morphology across loading cycles without progressive damage accumulation or structural collapse. During compression, the porous structure shrank along the compressive stress and radially buffered the external strain by elongating in the lateral direction, evidenced by increments of compressed lateral pore size. Under 50% compressive strain for 100 cycles, HPMP retained consistent compressive profiles with 94.6% maximum stress retention and elevated Young's modulus (Figure 6F). The robust compressive performance was not limited to cubic constructs but extended to semi‐annular printed constructs, which maintained bioprinting structural integrity under repetitive mechanical loading (Movie S1). The high reversible elasticity of HPMP may be attributed to microscale porous architecture, which combines structural resilience with elastomeric behavior [19, 45].

When detachment after complete compression testing, the HPMP was adhesive between the testing probe and testing platform, and the cyclic compressing stress‐time curves of HPMP exhibited two resiliencies in every cycle, potentially revealing distinct adhesion events (Figure S21A). Accordingly, we hypothesize that our HPMP possesses superior adhesive properties. To investigate the adhesive properties of HPMP, we initially performed a viscoelastic characterization of the material (Figure S22A). In comparison with pure GelMA, HPMP demonstrated significant stress relaxation behavior. This feature enables the material to accommodate continuous cyclic deformation, effectively dissipating localized stress concentrations, mitigating interfacial delamination, and ensuring durable and stable attachment. To further evaluate the adhesive capability of HPMP, shear‐tensile tests were conducted on HPMP adhered to tissue (Figure S22B, C). The result indicated that GelMA exhibited minimal adhesive efficacy, whereas HPMP at a 1:1 ratio of GelMA to PEO presented substantially superior tensile performance compared to ratios of 2:1 and 1:2. This enhancement is likely attributable to the hierarchical porous architecture of the material, which facilitates conformity to the microscale roughness of tissue surfaces, allowing rapid water absorption, swelling, and softening. Partial collapse of the pores may create a negative pressure zone between the hydrogel and the tissue, thereby increasing the effective contact area and enhancing overall adhesion performance [46, 47, 48, 49]. Moreover, the GelMA matrix of HPMP is rich in functional groups such as amide, amino, and carboxyl groups, which can form hydrogen bonds, electrostatic interactions, and hydrophobic associations with extracellular matrix proteins on myocardial tissue surfaces, further reinforcing the interfacial adhesion strength. To validate adhesion, we subsequently assessed interfacial bonding performance using porous hydrogel patches under physiologically relevant loading. The HPMP demonstrated robust cardiac adhesion resistant to mechanical dislodgement under shaking (Figure 6G; Movie S2). Adhesion assessments across diverse biological substrates further confirmed these properties. The HPMP stably approximated incised cardiac tissue (Figure S21B) and maintained adhesion of multiple organ specimens to glass slides for >6 h under inverted conditions (Figure 6H). To further validate its adhesion in physiological tissue, we took on the challenge in the beating heart with HPMP. The HPMP maintained stable adhesion with the cardiac surface during myocardial beating (Movie S3). These results establish that HPMP's hierarchically porous architecture confers excellent mechanical capability and long‐term tissue adhesion. This microgel‐based platform may advance regenerative strategies by mitigating patch detachment while accommodating physiological contractions.

### 2.5 Biological Functions of HPMP

**FIGURE 7:**
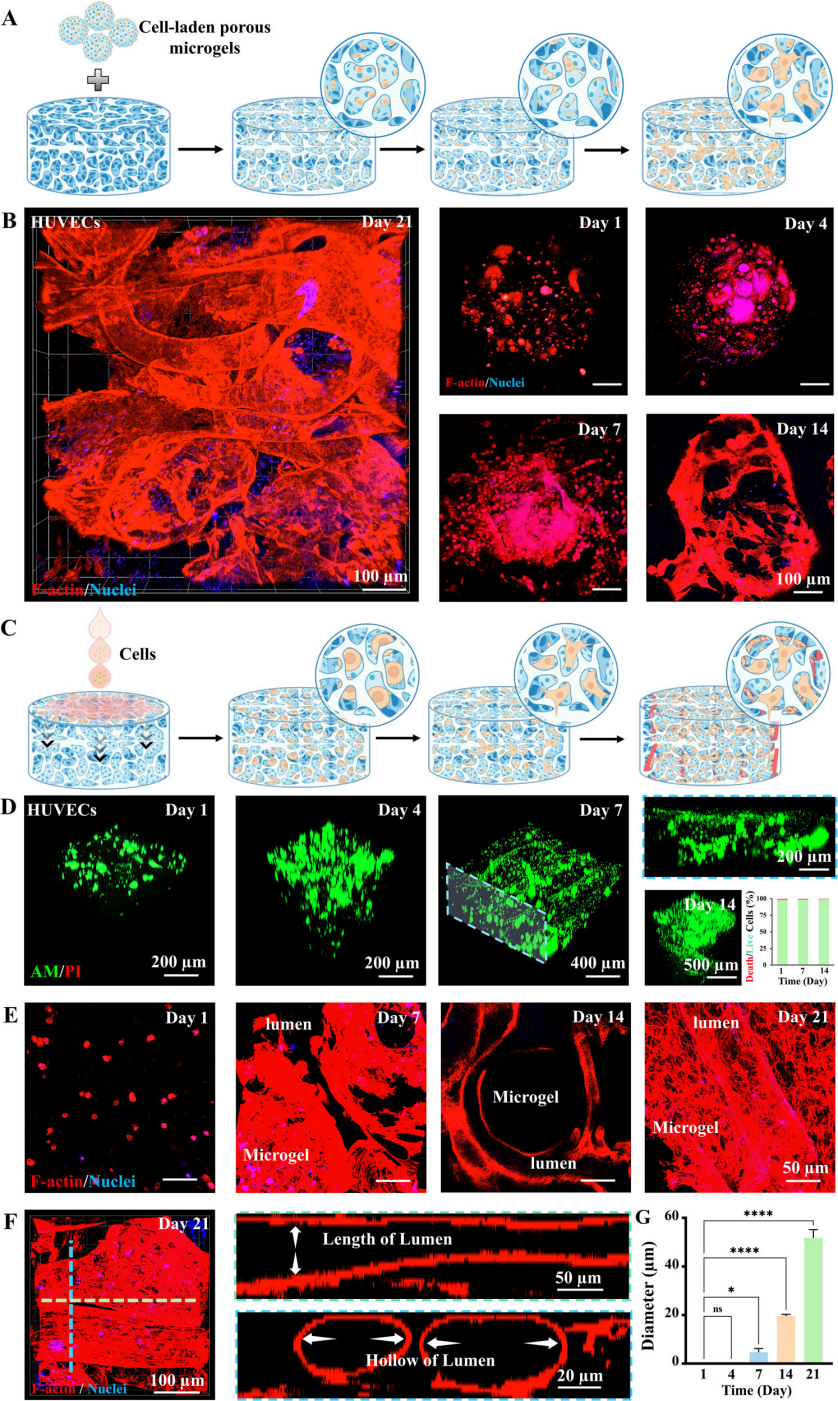
Cellular Behaviors and Vascularization in the Hierarchically Porous Hydrogel. (A) Schematic illustration of cell‐ladened HPMP; (B) Fluorescence images of the HPMP with HUVECs‐ladened porous microgels after 1, 4, 7, 14, 21 days culture stained for F‐actin (red), and nuclei (blue); (C) Schematic illustration of vascularization by cell seeded on the surface of HPMP; (D) Fluorescence images of Live/Dead staining for the HPMP after 1, 4, 7, 14, 21 days culture and quantification results of cell viabilities; (E) Fluorescence images of the cell seeded HPMP after 1, 4, 7, 14, 21 days of culture stained for F‐actin (red), and nuclei (blue) and DAPI; (F) Fluorescence images of the cell seeded HPMP by vertical section on the x‐axis (green) and y‐axis (blue); (G) Quantification results of lumens diameter over 21 days culture, n = 5; one‐way ANOVA; ^*^ *P* < 0.05, ^**^ *P* < 0.01, ^***^ *P* < 0.001, ^****^ *P* < 0.0001. Data are presented as mean values ± SDs (compared with the respective control groups of day 1).

The cell migration in engineering repair patches is crucial for repairing irreversibly damaged tissues [50]. To evaluate cellular behaviors in HPMP, we bioprinted constructs using HUVEC‐laden porous microgel bioinks and maintained cultures for 21 days (Figure 7A). HUVEC viability within HPMP was confirmed by Live/Dead assays (Figure S23). Immunofluorescence staining of F‐actin was used to verify the migration and morphology of HUVECs. The cells exhibited a sequent organized progression, characterized by sprouting by 4 days, cellular migration by 7 days, subsequently established vascular‐like lumens by 14 days, and ultimately formation with tightly interconnected networks by 21 days (Figure 7B). CD31 labelling further demonstrated functional maintenance, showing gradually increasing expression level before 7 days and culminating in well‐distributed and tightly luminal structures by 21 days (Figure S24). These results revealed that the HPMP architecture maintains bio/cytocompatibility and biological functionality while enabling cellular migration from microgels into bulk constructs. Furthermore, functional maintaining expression speculate that HPMPs suggests the potential vascularization in regenerative applications.

The establishment of functional vascular networks is essential for engineered tissue survival and function, as it enables critical nutrient/waste exchange and hemodynamic perfusion [51, 52]. To evaluate the vascularization capacity of HPMP scaffolds, HUVECs were seeded on cell‐free constructs and cultured for 21 days (Figure 7C). HUVECs infiltrated from superficial layers into HPMP via hierarchical porous structure within 4 days, progressing to lumen formation via Live/Dead assays by day 7 (Figure 7D). To further confirm vascularization within HPMP, F‐actin and CD31 staining revealed preliminary lumens inside HPMP at day 7, subsequent migration along the channels with porous microgels and porous network after 14 days, and tight vascularization in the entire hierarchical architecture after 21 days (Figure 7E; Figure S25A). These lumens exhibited progressive CD31 expansion and elongation with distinct closed luminal structures by 21 days (Figure 7F; Figure S25B–D). Longitudinal fluorescence images further demonstrated the temporal progression of hollow diameter of luminal structures from 20.31 ± 0.71 µm at day 14 to 51.73 ± 3.40 µm at day 21 with closed annular configurations (Figure 7G). These results indicate that the hierarchical porous structure of the HPMP supports external endothelial cell infiltration, migration, and vascularization, providing an advanced tissue regeneration platform.

### 2.6 Functional Assessments of Microgel‐Based Hydrogel Patch in Rat Models of MI

Cell‐based therapies utilizing functional cardiomyocytes have emerged as a promising therapeutic strategy for myocardial repair. However, these approaches face two fundamental challenges: (1) limited cell survival and proliferation in the hostile post‐ischemic microenvironment characterized by oxidative stress and inflammation, and (2) difficulties in achieving functional synchronization of transplanted cardiomyocytes with host tissue [53, 54]. Engineered biomaterial scaffolds could reconstruct physiological microenvironments to enhance cell‐based therapeutic efficacy. However, current cardiac patches often lack effective cell delivery and vascularization while requiring surgical suturing for stabilizing the scaffold, which risks tissue adhesion [55, 56]. Thus, developing multifunctional cardiac patches that concurrently achieve myocardial regeneration, functional vascularization, and anti‐adhesion presents a formidable translational challenge in cardiac tissue engineering. Given above established advantages of microgel‐based systems, our fabrication strategy is expected to provide a potential biomedical platform for structural and functional tissue regeneration.

**FIGURE 8:**
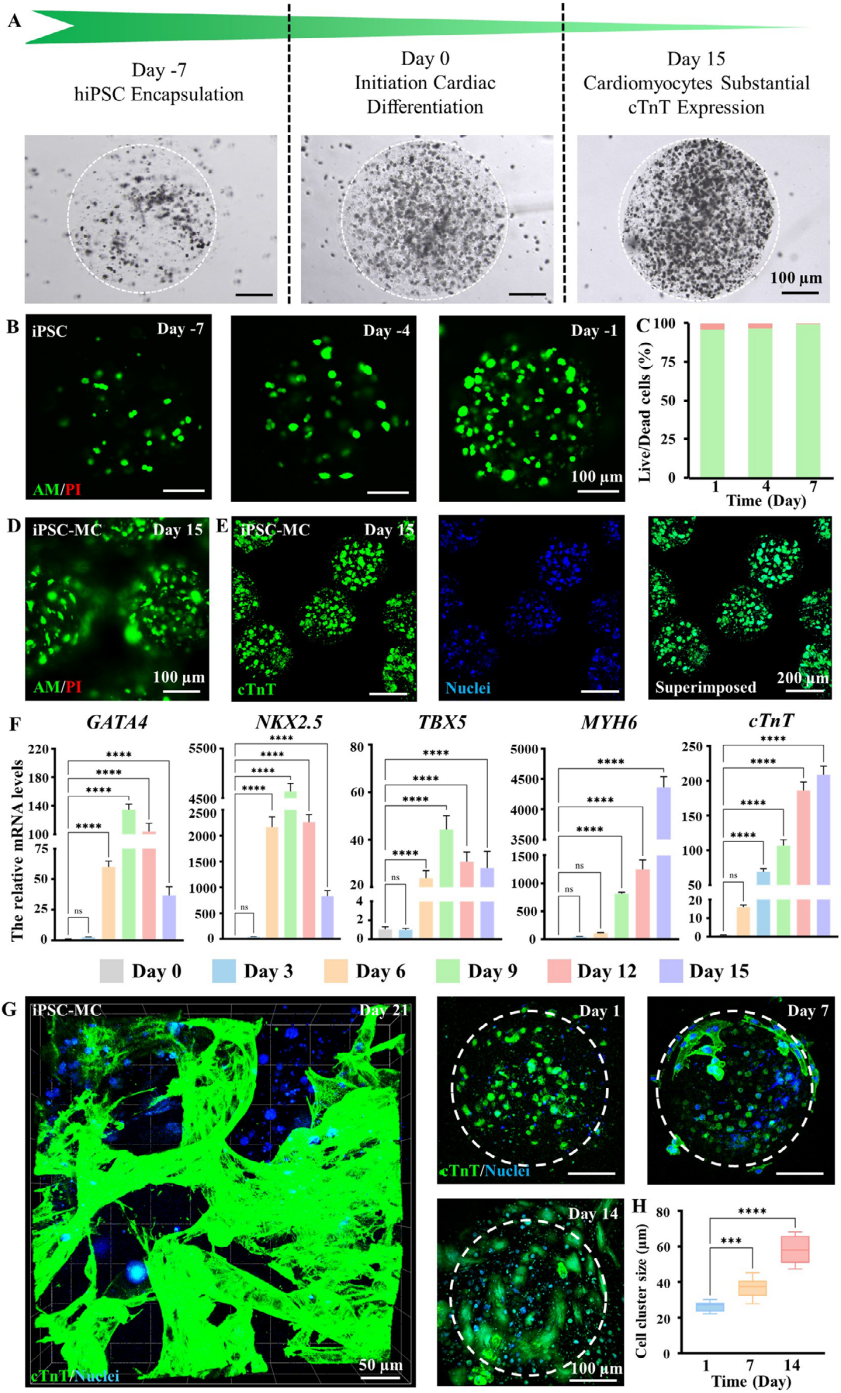
Cellular Behaviors and Differentiation of iPSC‐ladened Porous Microgels. (A) Process schematic and micrographs of iPSC‐ladened porous microgel culture and cardiac myocyte differentiation; (B) Fluorescence images of Live/Dead staining for iPSC‐ladened porous microgel culture over 7 days; (C) Quantification results of Live/Dead assay over 7 days culture; (D) Fluorescence images of Live/Dead staining for iPSC‐ladened porous microgel differentiation after 15 days; (E) Fluorescence images of iPSC‐ladened porous microgel differentiation after 15 days of culture stained for cTnT (green), and nuclei (blue); (F) Quantification results of the relative mRNA expression levels of cardiac markers (GATA4, NKX2.5, TBX5, MYH6 and cTnT) over the 15 days differentiation by qRT‐PCR, n = 4; one‐way ANOVA; ^*^ *P* < 0.05, ^**^ *P* < 0.01, ^***^ *P* < 0.001, ^****^ *P* < 0.0001. Data are presented as mean values ± SDs (compared with the respective control groups of day 0); (G) Fluorescence images of iPSC‐MC porous microgel within HPMP after 1, 7, 14, 21 days stained by cTnT (green), and nuclei (blue). (H) Quantification results of cellular spreading sizes in iPSC‐MC HPMP, n = 10; one‐way ANOVA; ^*^ *P* < 0.05, ^**^ *P* < 0.01, ^***^ *P* < 0.001, ^****^ *P* < 0.0001. Data are presented as mean values ± SDs (compared with the respective control groups of day 1).

To engineer myocardial repair patches, we fabricated cardiac engineering microgels using iPSCs and evaluated their cardiomyocyte differentiation potential. iPSC‐laden porous microgels were cultured for 7 days, followed by 15 days of directed cardiomyocyte differentiation (Figure 8A). The iPSC in porous microgels maintained >90% viability during initial culture (Figure 8B,C), with sustained high viability throughout cardiac differentiation (Figure 8D). Cardiac troponin T (cTnT, the cardiac cell marker protein) staining was performed at day 15 to confirm successful iPSC differentiation. As shown in Figure 8E, the cells exhibited robust expression of cTnT in porous microgels after cardiac differentiation. To investigate the progressive differentiation, we extracted cells from iPSC‐laden porous microgels at distinct time points for systematic evaluation with cardiac markers. Controlled microgel dissolution using ethylenediaminetetraacetic acid (EDTA)—an established divalent cation chelator often employed to disrupt Alg‐Ca^2^^+^ crosslinking—enabled the extraction of live cells (Figure S26) [57, 58, 59]. Quantitative reverse transcription polymerase chain reaction (qRT‐PCR) analysis of collected cells demonstrated stage‐specific upregulation of cardiac transcription factors during 15 days of iPSC differentiation. Early‐stage cardiac markers (GATA4, NKX2.5, TBX5) peaked at day 9, while late‐stage markers (MYH6 and cTnT) maximized at day 15 (Figure 8F). These results indicated the successful establishment of cardiomyocyte differentiation micro‐units by iPSC‐laden porous microgels. For the subsequent application of myocardial repair, we cultured HPMP scaffolds with iPSC‐derived cardiomyocytes (iPSC‐CMs) porous microgels for 21 days. iPSC‐CMs migration initiates at day 7, with progressive cellular spreading from the microgels. Subsequently, substantial iPSC‐CMs cellular aggregation exhibited dense and functional distribution in the interconnected porous network of HPMP at day 21 (Figure 8G,H). These results demonstrate HPMP to facilitate cardiomyocyte migration while maintaining functionality, providing an advanced delivery platform for myocardial repair.

Despite the excellent adhesion of HPMP with tissue, this property risks postoperative thoracic adhesions that may further injure vulnerable myocardium [60]. To address this, we engineered a Janus‐adhesive hierarchical porous microgel‐based hydrogel patch (JHPMP) via multi‐material bioprinting of microgel‐based bioinks and hyaluronic acid methacrylate (HAMA), exhibiting asymmetric adhesion functionality. To evaluate the feasibility of multilayer printing, we used DLP printing to fabricate a cylinder with well‐defined multilayer constructs (Figure S27A). The stable stratified junction of HPMP and HAMA was successfully observed (Figure S27B). JHPMP also maintains distinct reversible compressibility, with no significant structural break or deformation after 100 compression cycles (Figure S27C, D; Movie S4). To assess cellular behavior, we employed JHPMPs incorporating H9C2‐laden microgels in the porous layer and acellular HAMA. As illustrated in Figure S27E, F, the cells preferentially migrated toward the porous structural side of the junction after 14 days.

**FIGURE 9:**
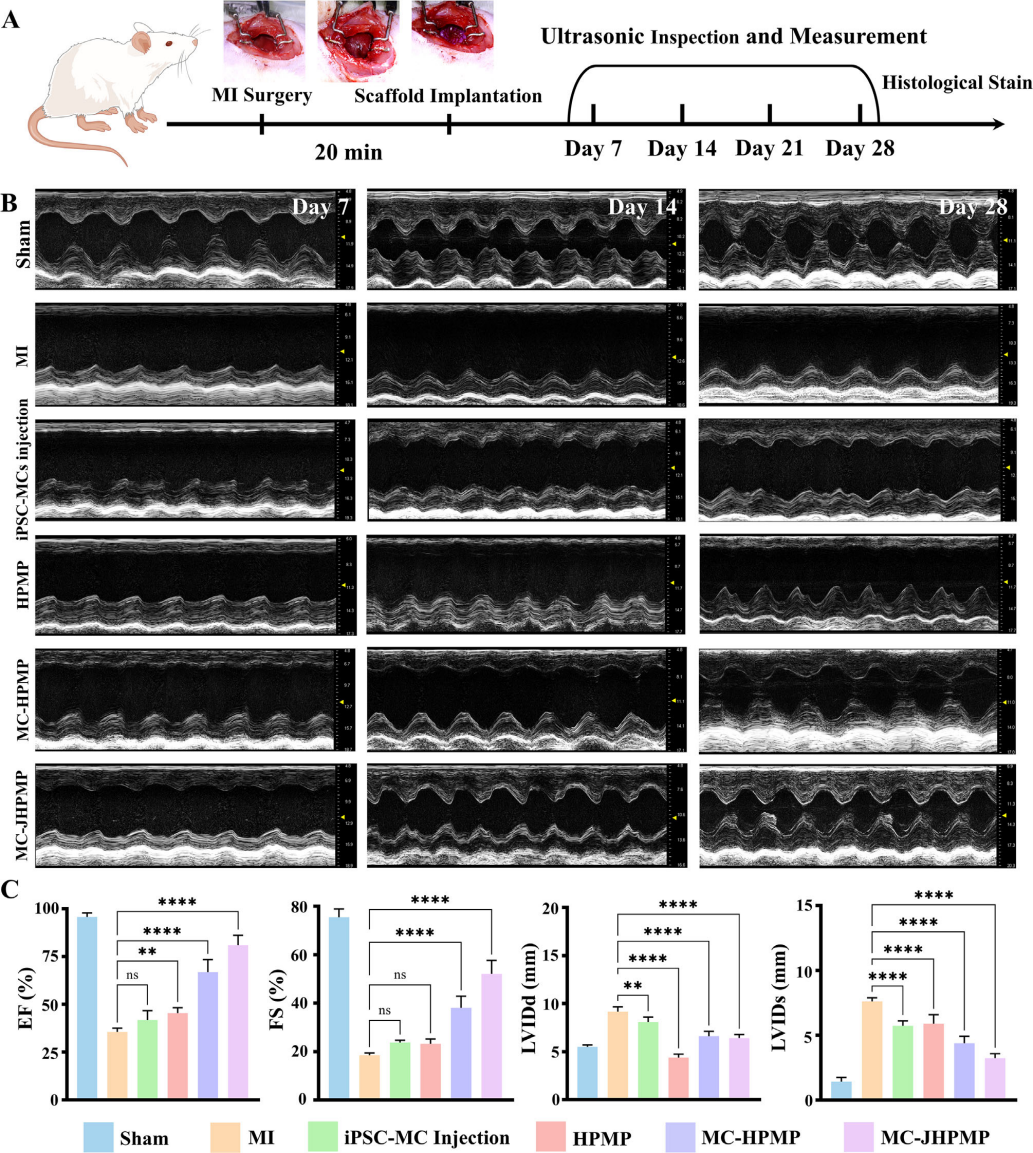
Cardiac Function in Rats with MI via Hierarchically Porous Hydrogel with iPSC‐MCs. (A) Schematic of the experimental process with cardiac function and histogical assessment after MI; (B) Representative echocardiography images of each group at 7, 14, and 28 day; (C) Quantitative analysis at 28 day end point, n = 5; one‐way ANOVA; ^*^ *P* < 0.05, ^**^ *P* < 0.01, ^***^ *P* < 0.001, ^****^ *P* < 0.0001. Data are presented as mean values ± SDs (compared with the respective MI groups).

Since the favorable cellular and mechanical properties of microgel‐based fabrication, we evaluated its therapeutic efficacy in a rat MI model established by left anterior descending coronary artery ligation (Figure 9A) [61, 62]. To evaluate the efficacy of myocardial infarction repair, we first assessed the heart function of rats via echocardiography within 28 days. As shown in Figure 9B, bare contraction occurred in the left ventricular anterior wall in the MI and HPMP groups, and smaller contraction waves were observed in the iPSC‐MCs injection group, compared with the sham group. In contrast, the MC‐HPMP and MC‐JHPMP groups showed significant contractile activity of the left ventricular anterior wall. The cardiac function at the day 28 end point was evaluated by analyzing typical echocardiography parameters, including ejection fraction (EF), fractional shortening (FS), left ventricular internal dimension in systole (LVIDs), and left ventricular internal dimension in diastole (LVIDd). Among them, both EF and FS were significantly enhanced in the MC‐HPMP and MC‐JHPMP groups compared to the MI group, while significantly decreased LVIDs and LVIDd, indicating the cardiac function had improved (Figure 9C).

We reopened the thoracic cavities after 28 days post‐implantation to further evaluate the asymmetric adhesion performance of JHPMP in vivo (Figure S28). Gross morphology revealed severe mediastinal adhesions in the HPMP and MC‐HPMP groups, with cardiac tissue fused to the chest wall. Conversely, MC‐JHPMP exhibited firm adhesion to the ventricular base of apex cordis without synechia of the thorax, while permitting complete cardiac elevation without causing thoracic structural deformation. The possible reason for this phenomenon is that JHPMP not only effectively prevents adhesion through its superior physical barrier properties [63] but also incorporates active biological regulation [64]. Specifically, the HAMA faces the thoracic cavity, while the HPMP is oriented toward the heart. The HAMA, with a smooth surface and high biocompatibility, physically prevents direct contact between damaged tissues and surrounding organs. This design simultaneously inhibits the migration of iPSC‐MCs toward the thoracic cavity, and also eliminates the risk of additional adhesion caused by cell diffusion, securely retaining therapeutic cells in their designated locations and minimizing cell leakage.

**FIGURE 10:**
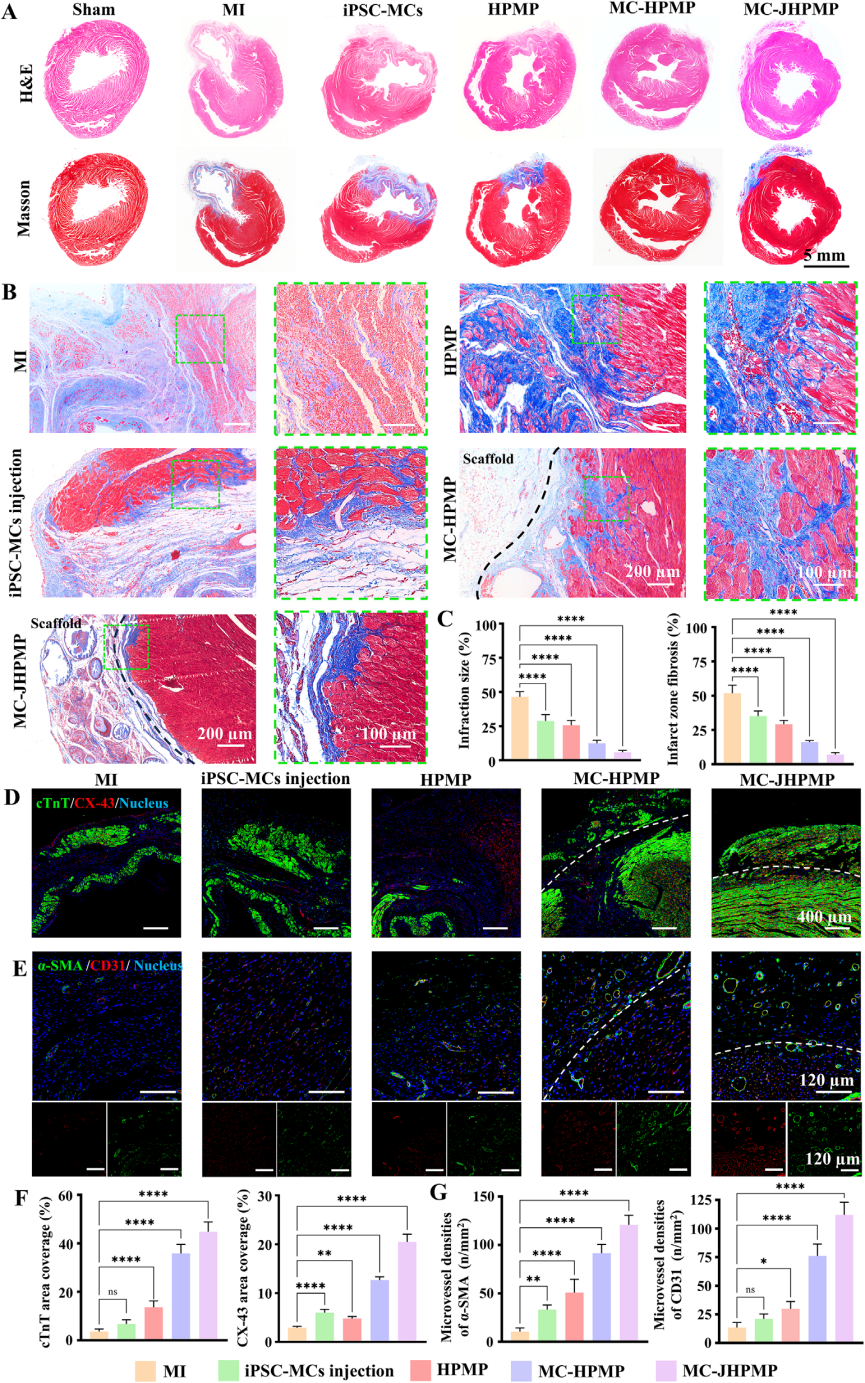
Histochemical Assessment of Regeneration and Vascularization Myocardial Infarction in Rats after 4 weeks. (A) H&E and Masson trichrome staining of hearts in different group; (B) Representative images in the infarct edge zones of Masson trichrome images; (C) Quantitative analysis of the infarct area and the collagen fibrosis in infarct area; (D) Immunofluorescence images in infarct area with cTnT (green) and CX‐43 (red), with white dashed lines indicating the border locations of the patches; (E) Immunofluorescence images in infarct area with α‐SMA (green) and CD31 (red), with white dashed lines indicating the border locations of the patches; (F‐G) Quantitative analysis of cTnT, CX‐43, α‐SMA and CD31, n = 5; one‐way ANOVA; ^*^ *P* < 0.05, ^**^ *P* < 0.01, ^***^ *P* < 0.001, ^****^ *P* < 0.0001. Data are presented as mean values ± SDs (compared with the respective MI groups).

To assess therapeutic efficacy, we characterized the heart of each experimental group via histological examination of cardiac tissue at the day 28 endpoint, staining by hematoxylin‐eosin (H&E) and Masson trichrome (Figure 10A; Figure S29). Both MI and HPMP groups exhibited disorganized wavy fibers of myocytes in the infarct core and indistinct cellular margins at the periphery. Notably, the MI group displayed myocytolysis as an additional ischemic change, indicating cardiomyocyte apoptosis and impaired regeneration. The presence of wavy myofibers and isolated cardiomyocytes in the infarct core and border zones of the iPSC‐MCs injection group suggested poor integration between delivered exogenous cells and host myocardium. In contrast, MC‐HPMP and MC‐JHPMP groups showed well‐defined cellular morphology, with MC‐JHPMP further displaying most cardiomyocytes with striation and vesicular oval nuclei (Figure S29E, F). This indicated that the significant therapeutic effect was in blocking collagen fiber accumulation after treatment with microgel‐based hydrogel patches. Furthermore, to further assess cellular architecture and collagen deposition, Masson trichrome staining (blue indicating collagen deposition) was used to demonstrate the situation of fibrotic remodeling. Comparatively, both MC‐JHPMP and MC‐HPMP groups demonstrated reduced fibrotic burden, while the MC‐JHPMP group displayed fibrosis levels equivalent to the sham group (Figure 10A,B). The quantitatively analysis demonstrated the smallest infarct zone of cardiac tissue was observed in MC‐JHPMP group treated compared to MI group (Figure 10C), the infarct area with 45.43 ± 4.54% (MI group), 30.53 ± 3.20% (cell group), 25.78 ± 4.49% (HPMP group), 12.33 ± 2.83% (MC‐HPMP group), 6.10 ± 1.60% (MC‐JHPMP group). Further, a marked reduction in fibrotic area in MC‐HPMP and MC‐JHPMP groups compared to the MI group, with the latter exhibiting fibrosis of collagenous regions by blue‐stained in all MI models, and average fractions of the collagen fibrosis area to total left ventricle with 50.38 ± 6.28% (MI group), 35.20 ± 2.54% (cell group), 27.41 ± 2.15% (HPMP group), 16.29 ± 1.56% (MC‐HPMP group), 7.63 ± 1.72% (MC‐JHPMP group).

To further evaluate therapeutic efficacy, we assessed cellular gap junction formation and angiogenesis by immunofluorescence co‐staining of cTnT and connexin 43 (Cx43), critical for intercellular communication via gap junctions between cardiomyocytes. The MI group exhibited the absence of gap junction formation with no discernible linear distribution (Figure 10D, F). Although the iPSC‐MCs injection group showed increased gap junction formation compared to the MI group, these junctions were localized predominantly within the injected cell clusters rather than the infarcted myocardium area. In microgel‐based hydrogel patch groups, both MC‐HPMP and MC‐JHPMP groups showed enhanced gap junction formation at cardiomyocyte interfaces within the patch and myocardium integration zones. Notably, MC‐JHPMP exhibited stronger fluorescence by cTnT and Cx43 co‐localization. To confirm the angiogenic potential, we assessed the vessels via α‐smooth muscle actin (α‐SMA) and CD31 immunostaining. As revealed in Figure 10E, the MI group had sparse vascular networks, whereas the MC‐HPMP and MC‐JHPMP groups showed dense, interconnected neovessels. The density of microvessels in MC‐JHPMP surpassed all groups, confirming that microgel‐based patches play a critical role in the promotion of functional angiogenesis (Figure 10G). In addition, the biocompatibility assessment of transplanted HPMP via H&E staining of major organs (lung, liver, spleen, and kidney) revealed no pathological abnormalities or inflammation at 28 days (Figure S30). The microgel‐based modular fabrication strategy exhibits significant biomedical potential for myocardial repair. Engineered microgels with stem cell differentiation enable the fabrication of Janus unilateral adhesion patches, mitigating secondary injury and tissue adhesion associated with patch implantation. This platform also facilitates efficient cardiomyocyte delivery while promoting neovascularization, thereby providing excellent physicochemical microenvironments in MI. Ultimately, integration of structural reconstruction with functional restoration enables coordinated cardiac repair post‐infarction.

## 3 Conclusion

Tissue engineering and regenerative medicine target structural and functional tissue regeneration, necessitating bio‐manufacturing platforms that integrate cellular survival, proliferation, differentiation, and spatially organized growth. Although 3D‐bioprinted hydrogel scaffolds enable precise architectural control, conventional bulk hydrogels still confront limitations: nanoscale polymer networks restrict nutrient diffusion and impair cellular migration, while extrusion‐based printing induces shear‐mediated cellular damage that compromises encapsulated cell viability. Critically, efficient regeneration requires in vivo restoring vascularized functional tissue, not non‐functional fibrotic repair.

In this work, we developed a novel modular bioprinting strategy constructing hierarchically porous microgel‐based patches, wherein gas‐shearing microfluidics and ATPS enable precision‐engineered porous microgels with tunable porosity and high cytocompatibility. These microgel units serve as modular bioinks, facilitating fabrication of HPMPs exhibiting exceptional compressibility, tissue adhesion, and cellular adaptability. In vitro evaluation demonstrated significant cell proliferation and migration within both porous microgels and HPMP constructs, specifically within the HPMP architectures with an interconnected porous structure facilitated vascular network formation. To validate the biomedicine potential in tissue regeneration, we applied this strategy to establish a cardiac tissue regeneration engineering patch for MI therapy. We engineered iPSC‐cardiomyocyte‐laden microgels and printed JHPMP to address the challenge of the beating heart's unilateral adhesion. Our study demonstrates that the JHPMP with iPSC‐derived cardiomyocyte‐laden microgels effectively mitigates beating heart‐thoracic adhesion, significantly promoting cardiac tissue regeneration and functional neovascularization. The anti‐adhesive physical barrier by JHPMP also functions as a myocardial‐side targeted therapeutic mechanism for cardiomyocyte delivery. Meanwhile, the porous structure of JHPMP potentially augments the paracrine effects of the transplanted cells, facilitating a more sustained and efficient interaction with the host tissue. This provides a more stable and regenerative microenvironment for transplanted myocardial cells and new blood vessels to thrive, thereby achieving a more durable and effective repair platform. Thus, modular bioprinting directly addresses the structural limitations of conventional microgels, reconciling the cytoprotection during fabrication with the post‐implantation ECM remodeling for effective tissue repair.

The microgel‐based modular fabrication strategy establishes multifunctional medicine platforms through hierarchically porous microgel bioinks, where tunable microgel preparation and modular 3D bioprinting synergistically enhance biomimetic functionality. By employing discrete cell‐laden microgels as building blocks, this approach resolves the functional tissue integration and regeneration in vivo through interconnected porosity mimicking native ECM architecture, enabling cellular infiltration and vascular network formation. Crucially, modular pores provide microenvironments for cell migration that allow encapsulated cells to transmigrate and remodel nascent ECM, enabling functional host integration rather than passive encapsulation. In conclusion, the microgel‐based platform establishes a transformative tissue engineering paradigm for tissue regeneration, with demonstrated success in myocardial repair and extensible potential for osteochondral reconstruction and flap regeneration, etc., effectively bridging critical gaps between foundational research and clinical translation.

## 4 Experimental Section/Methods

### 4.1 Materials

The following materials were used in this study: porcine skin gelatin (Sigma, Type A, ∼300 g bloom, average Mw = 90 kDa), Methacrylic anhydride (MAA), alginate (Aladdin, S100128), dextran (Aladdin, D490149‐750K), PEO (Sigma‒Aldrich, 182001, 300 K), fluorescent polystyrene nanoparticles (Dae), DMEM (Gibco), endothelial cell medium (ECM Sciencell 1001), PSCeasy and CardioEasy (Cellapy), Calcein/PI staining kit (C2015M, Beyotime), PrestoBlue HS Staining Kit (Thermo Fisher, P50200), SYBR Green Pro Taq HS for qPCR (agbio, AG11701), Anti‐Cardiac Troponin T Rabbit pAb, Anti‐alpha smooth muscle Actin Rabbit pAb and Anti‐Connexin 43/GJA1 Rabbit pAb (Servicebio).

### 4.2 Gas‐shearing Microfluidic Equipment

The gas‐shearing microfluidic platform is mainly composed of four parts: an electronic syringe pump delivering the ATPS solution; a nitrogen gas supply cylinder regulated by a flowmeter to control the nitrogen flow rate; a coaxial needle fabricated by nesting a 27‐gauge core needle within an 18‐gauge shell needle, with an additional 14‐gauge needle fixed to the shell for gas delivery; and a microgels collection bath. All joints were sealed with tubes.

### 4.3 Preparation of Porous Microgel

A hydrogel precursor was prepared by dissolving sodium alginate (Alg) and dextran (Dex) in PBS as the continuous phase. This continuous phase solution was combined with 2% poly(ethylene oxide) (PEO) to form the hydrogel pre‐mixture. A microgel collection bath was prepared by dissolving poly(ethylene glycol) (PEG) in 2% Ca^2^^+^ solution. The hydrogel pre‐mixture (aqueous phase, 1.0 mL·h^−^^1^) and nitrogen gas (0.8 L·min^−^^1^) were co‐extruded through the coaxial needle, generating gas‐sheared droplets that solidified in the receiving bath. After 5 min incubation, microgels were washed three times with PBS via sedimentation and supernatant removal.

### 4.4 Characterization of Porous Structures

The fluorescent hydrogel precursor was prepared using Alg‐Dex with red fluorescent nanoparticles and PEO with green fluorescent nanoparticles. Following the fabrication protocol for porous microgels, time‐point observations were conducted immediately upon formation.

### 4.5 Synthesis of GelMA

Gelatin methacryloyl (GelMA) was synthesized by dissolving 10 g of porcine skin gelatin in 100 mL PBS at 40°C, followed by dropwise addition of 8 mL methacrylic anhydride under continuous stirring (240 rpm for 2 h). The solution was diluted with 100 mL pre‐warmed PBS (50°C) and mixed for 10 min. Dialysis was performed using a membrane (pre‐inspected for defects) submerged in 5 L distilled water at 40°C for 5 days, with water replacement every 12 h and membrane inversion (5‐6 times per change). Post‐dialysis, the GelMA was filtered (0.8 µm) at 40°C, aliquoted into 50 mL tubes, and freeze‐dried for 5 days before storage at 4°C.

### 4.6 Fabrication of HPMP Bioink

The hydrogel phase was prepared by dissolving lyophilized GelMA and 0.5% lithium phenyl‐2,4,6‐trimethylbenzoylphosphinate (LAP) in PBS to achieve 2.5–15% GelMA concentrations. Sacrificial phases consisted of 1.6% polyethylene oxide (PEO) mixed with porous microgels (1:1 ratio). Bioinks were formulated by blending sacrificial and hydrogel phases at volumetric ratios of 3:2 to 2:3.

### 4.7 3D Bioprinting of HPMP

For extrusion‐based Bioprinting, bioinks were loaded into 5 mL cartridges, equilibrated at the GelMA‐PEG‐PMG was pre‐cooled at 4°C for 5 min, printing maintained at 10°C, and printed using a 3D Bio‐Architect Sparrow system (70 mm·min^−^^1^, 0.1‐0.25 kPa pneumatic pressure, 10–15°C). Printed constructs were crosslinked under UV (0.5 W/cm^2^, 30 s), rinsed with 37°C PBS, and mechanically tested. For digital light processing (DLP) Bioprinting, the Regenovo 3D Bio‐Architect Parrot was employed for DLP, and bioinks were maintained at 37°C during layer‐by‐layer printing (3 s/layer, 0.5–2.5 W/cm^2^ UV). After printing, constructs underwent identical PBS washing and characterization protocols.

### 4.8 Mechanical Characterization

For rheological characterization of bioink, the linear viscoelastic region was determined via amplitude sweep tests at 1 Hz and 37°C, confirming 1% strain as suitable. Temperature sweeps (0‐40°C at 2°C/min) and frequency sweeps (0.1–100 rad/s) were then performed at 1% strain to characterize the moduli, using automated protocols for consistent data acquisition. Microgels and HPMP were subjected to uniaxial compression (10–50% strain, 10 s hold) or cyclic compression (50% strain, 50–1000 cycles) via an SMS texture analyzer. The samples were preloaded to 0.01N at a strain rate of 1 mm/min, with the size of 78.00 mm2 maintained in a room temperature environment. For the compression stress relaxation test, the HPMP was compressed to 50% of its original thickness and maintained at this constant strain. The stress of sensors was recorded as a function of time at room temperature. For the lap‐shear tensile testing, the hydrogel was adhesively bonded between two muscle tissue specimens. A tensile sensor was then employed to stretch the muscle tissue at a constant rate of 50 mm min^−^^1^ until fracture occurred within the hydrogel interlayer. The tensile strength was defined as the stress recorded at the point of fracture.

### 4.9 Cell Culture

For Hela (iCell, iCell‐h088), H9C2 (iCell, iCell‐r012), complete DMEM medium (10% FBS, 1% penicillin‐streptomycin), and for HVUECs (iCell, iCell‐h110), the ECM was prepared under sterile conditions. Cells were maintained until 90% confluency, followed by PBS washing, trypsinization, and centrifugation (900 rpm, 3 min). Resuspended cells were subcultured at a 1:2 ratio in fresh medium. Human induced pluripotent stem cells (iPSCs, Cellapy, CA1002008) were cultured using standardized protocols. For cell recovery, 3 mL/well of iPSC coating solution was added to 6‐well plates and incubated overnight. After removing the coating solution, iPSC recovery medium containing 1 µL/mL Y27632 was prepared. Thawed iPSC cryopreservation aliquots were slowly supplemented with 2 mL iPSC medium followed by centrifugation at 1000 rpm for 3 min. The supernatant was discarded, and the cell pellet was resuspended in recovery medium before transfer to pre‐treated plates.

### 4.10 Fabrication of Cell‐Laden Porous Microgel and HPMP

For cell‐laden porous microgels, confluent cells were trypsinized, counted, and adjusted to 1 × 10^6^ cells/mL. The cell suspension was centrifuged, mixed with 2% PEO, and processed using the aforementioned microgel protocol. Microgels were cultured in DMEM with medium replacement every 48 h. For HPMP culturing, bioink with cell‐laden porous microgels was bioprinted into HPMP, cultured with medium replacement every 48 h. Viability assessment (Live/Dead staining) and immunostaining (CD31/F‐actin) were performed at 1, 4, 7, 14, and 21 days using established protocols.

### 4.11 Vascularization of HPMP

Cell‐free porous microgels were bioprinted into HPMP, and seeded HUVECs in HPMP cultured in ECM, with medium replacement every 48 h. Viability assessment (Live/Dead staining) and immunostaining (CD31/F‐actin) were performed at 1, 4, 7, 14, and 21 days using established protocols.

### 4.12 iPSC‐Laden Porous Microgels Culture and Differentiation

For microgel encapsulation, dissociated iPSCs were resuspended in 2% polyethylene oxide (PEO) solution until achieving appropriate cell cluster dispersion, followed by porous microgel fabrication using established methods. Cardiac differentiation within microgels proceeded through sequential media changes. After 48 h incubation in Cardiac Differentiation Medium I at 37°C with 5% CO_2_, microgels were washed with PBS and transitioned to Differentiation Medium II for an additional 48 h. Subsequent replacement with Differentiation Medium III occurred every 48 h, with regular medium refreshment until cTnT immunostaining analysis.

### 4.13 PCR Analysis of iPSC‐Laden Porous Microgels

RNA extraction from microgel‐encapsulated cells at specified time points (days 0, 4, 7, 10, 14, 21) involved alginate dissolution using 0.01 M EDTA solution. Following centrifugation at 1000 rpm for 3 min and PBS washing, cell pellets were lysed with Trizol reagent. Phase separation was achieved through chloroform addition and centrifugation at 12 000 rpm for 15 min at 4°C. RNA precipitation using isopropanol was followed by ethanol washing and dissolution in DEPC‐treated water. RNA concentration was quantified spectrophotometrically before storage at −80°C. PCR analysis of cells encapsulated within porous microgels was conducted through a three‐stage molecular workflow. Genomic DNA removal was initiated by assembling a 10 µL reaction mixture on ice containing processed RNA solution, 2 µL gDNA elimination reagent, 2 µL 5 × buffer, and RNase‐free water. The mixture underwent brief centrifugation followed by thermal cycling for residual DNA degradation before storage at −20°C. Complementary DNA synthesis employed the Evo M‐MLV RTase system, combining 10 µL processed gDNA‐free sample with 1 µL reverse transcriptase enzyme mix, 1 µL PT primer cocktail, 4 µL 5× reaction buffer, and 4 µL RNase‐free water. The reverse transcription protocol was executed through programmed thermal cycling, with resultant cDNA aliquots preserved at ‐20°C. For quantitative analysis, cDNA templates were diluted to <100 ng/µL using nuclease‐free water. Real‐time PCR reactions were configured in 20 µL volumes containing 5 µL 2× SYBR Green master mix, 3.2 µL molecular‐grade water, 0.4 µL forward primer, 0.4 µL reverse primer, and 1 µL diluted cDNA template. Following brief centrifugation in a pre‐chilled microcentrifuge (3000 rpm), amplification profiles were generated using a calibrated real‐time PCR cycler with standardized thermal parameters. The primer sequences are listed in Table S1.

### 4.14 Immunostaining

For Live/Dead staining, calcein‐AM (1 µL in 1 mL PBS) and propidium iodide (PI, 1 µL in 1 mL PBS) were prepared. On days 1, 4, and 7, microgels were incubated with Calcein‐AM for 20 min, followed by PI for 10 min. Fluorescence imaging was performed using inverted and confocal microscopes. Microgels and HPMP were fixed with 4% paraformaldehyde (15 min), permeabilized with 0.5% Triton X‐100 (15 min), and blocked with 5% BSA (1 h). Primary antibodies (CD31/cTnT, 1:200 in 1% BSA) were applied overnight. After PBST washing, fluorescent secondary antibodies (1:500) and F‐actin (1:1000) were incubated for 1.5 h and 30 min, respectively. Nuclei were stained with Hoechst (5 min) and imaged. For cell proliferation assay, cell‐laden microgels were centrifuged at 400 g for 3–5 min at 4°C, followed by removal of the supernatant. The microgels were washed twice with PBS, each wash lasting 5 min. Subsequently, 1 mL of the CFDA‐SE working solution was added to the microgels, and the mixture was incubated at room temperature for 30 min. After incubation, the microgels were centrifuged, and the supernatant was discarded. The microgels were then washed twice with PBS. Finally, the microgels were observed under a fluorescence microscope for analysis.

### 4.15 Animals

Male Sprague‐Dawley (SD) rats (weight: 250 ± 20 g; specific pathogen‐free (SPF) grade) were obtained from Beijing Vital River Laboratory Animal Technology Co., Ltd. (Beijing, China; Certification No. SCXK 2021–0168). All animals were housed under standard SPF conditions (temperature: 22 ± 2  °C; humidity: 55 ± 10%; 12 h/12 h light/dark cycle) with ad libitum access to sterile food and water. Rats were acclimatized for at least 7 days prior to any experimental procedures. All animal experiments were approved by the Animal Ethics Committee of Guangzhou Medical University (China; Certification No. SYXK 2023‐0227, Project Approval No. GY 2024–548).

### 4.16 Animal Experiment Design and Methodology

The experimental design comprised five groups: a sham group and four experimental groups (n = 5). The sham group underwent thoracotomy with pericardial exposure followed by suturing without further intervention. We employed iPSC‐MC injection without porous microgels (as iPSC‐MC injection group) and cell‐free HPMP (as HPMP group), and iPSC‐MC‐laden porous microgels as bioink to fabricate cell‐laden HPMP (as MC‐HPMP group) and JHPMP (as MC‐JHPMP group) respectively, subsequently implanted into rat hearts feeding over 4 weeks. Myocardial infarction (MI) was induced in model groups by ligating the left anterior descending (LAD) coronary artery using 7‐0 silk sutures, confirmed by visible pallor in the LAD‐supplied myocardium, as follows: Surgical Procedures. The iPSC‐MC injection group received five intramyocardial injections (20 µL each, total 10^6^ cells) into peri‐infarct regions post‐MI induction. The HPMP group was treated with a cellular hydrogel adhered to the beating heart until stable fixation, while the MC‐HPMP and MC‐JHPMP groups received hydrogel loaded with iPSC‐derived cardiomyocytes (iPSC‐MCs) under identical conditions. JHPMP is composed of the porous and adhesive surface of HPMP and the smooth and non‐adhesive surface of HAMA, with HAMA facing the thoracic cavity and HPMP facing the heart.

### 4.17 Surgical Procedures

Male SD rats were anesthetized with a mixture of Zoletil (60 mg/kg) and xylazine hydrochloride (10 mg/kg) dissolved in PBS. Following tracheal intubation and mechanical ventilation, a left thoracotomy was performed at the third intercostal space to expose the heart. LAD ligation was executed 2–3 mm below the left auricle using a 5‐0 suture, with MI success validated by regional whitening of the ventricular wall. Post‐ligation, treatment groups received respective interventions before layered closure of thoracic layers. The sham group underwent thoracotomy without LAD ligation.

### 4.18 Echocardiographic Assessment

Transthoracic echocardiography was performed under isoflurane anesthesia at postoperative 7 days, 14, and 28. Left ventricular functional parameters, including ejection fraction (EF), fractional shortening (FS), end‐diastolic volume (EDV), and end‐systolic volume (ESV), were quantified.

### 4.19 Anti‐Adhesion Scoring Method

Scoring the degree of the anti‐adhesion experiment in vivo by semi‐quantitatively graded according to the following scheme: 0 is normal pleural space; 1 is one to three small adhesions in the pleural space; 2 is more than three scattered adhesions, but lung easily separated from chest wall; 3 is generalized scattered adhesions with areas where the lung can be separated from the chest wall only with difficulty; 4 is complete obliteration of the thoracic pace by adhesions.

### 4.20 Histopathological and Immunofluorescence Analysis

Hearts were harvested at day 28, fixed in 4% paraformaldehyde, and processed for paraffin sectioning (4 µm). Hematoxylin‐eosin (H&E) and Masson's trichrome staining evaluated tissue morphology and fibrosis. For immunofluorescence, sections were permeabilized with 0.5% Triton X‐100, blocked with 1% BSA, and incubated with anti‐cTnT, anti‐Cx43, anti‐α‐SMA, and anti‐CD31 primary antibodies overnight at 4°C, followed by species‐matched secondary antibodies. Nuclei were counterstained with DAPI. Digital slide scanning and ImageJ‐based quantification were employed for histological and fluorescence signal analysis.

### 4.21 Statistical Analysis

Quantitative data are expressed as mean ± SD. Intergroup differences were assessed using a two‐tailed unpaired Student's t‐test for pairwise comparisons or one‐way ANOVA with Bonferroni post‐hoc correction for multiple groups, using GraphPad Prism 9.5. Significance thresholds were set at ^*^ *P*<0.05, ^**^ *P*<0.01, ^***^ *P*<0.001, and ^****^ *P*<0.0001.

## Conflicts of Interest

The authors declare no conflict of interest.

## Supporting information




**Supporting File 1**: advs73705‐sup‐0001‐SuppMat.docx.

**Supporting File 2**: advs73705‐sup‐0002‐MovieS1.mp4.

**Supporting File 3**: advs73705‐sup‐0003‐MovieS2.mp4.

**Supporting File 4**: advs73705‐sup‐0004‐MovieS3.mp4.

**Supporting File 5**: advs73705‐sup‐0005‐MovieS4.mp4.

## Data Availability

The data that support the findings of this study are available in the Supporting Information of this article.
