# Supplementary material for: Microgel‐Based Hierarchical Porous Hydrogel Patch with Adhesion and Resilience for Myocardial Infarction
Source: Adv Sci (Weinh). 2026 Jan 5;13(16):e18646. doi: 10.1002/advs.202518646 (PMC13042884; doi:10.1002/advs.202518646)
Supplement: Supplementary file 1 — Supporting File 1: advs73705‐sup‐0001‐SuppMat.docx. [file ADVS-13-e18646-s002.docx]

Supporting Information

Microgel-Based Hierarchical Porous Hydrogel Patch with Adhesion and Resilience for Myocardial Infarction

*Ziyang Liu^1#^, Leyan Xuan^1#^, Yingying Hou^1,2#^, Ting Xie^1^, Jieting Li^1^, Junjie Cai^1^, Siyu Zhang^1^, Yingling Miao^1^, Ning Hou^1^, Gen He^1^, Maobin Xie^2^, Xiyong Yu^1^*, Mingen Xu^3^*, Guosheng Tang^1^**

Ziyang Liu, Leyan Xuan, Yingying Hou, Ting Xie, Jieting Li, Junjie Cai, Siyu Zhang, Yingling Miao, Ning Hou, Gen He, Xiyong Yu, Guosheng Tang

1Guangzhou Municipal and Guangdong Provincial Key Laboratory of Molecular Target & Clinical Pharmacology, the NMPA and State Key Laboratory of Respiratory Disease, School of Pharmaceutical Sciences, Guangzhou Medical University, Guangzhou 511436, China.

E-mail: guoshengtang@gzhmu.edu.cn, yuxycn@aliyun.com

Maobin Xie Yingying Hou

2The Fourth Affiliated Hospital of Guangzhou Medical University School of Biomedical Engineering Guangzhou Medical University Guangzhou 511436, P. R. China.

Mingen Xu

3School of Automation, Hangzhou Dianzi University, Hangzhou 310018, People’s Republic of China.

**Supplementary Figures:**


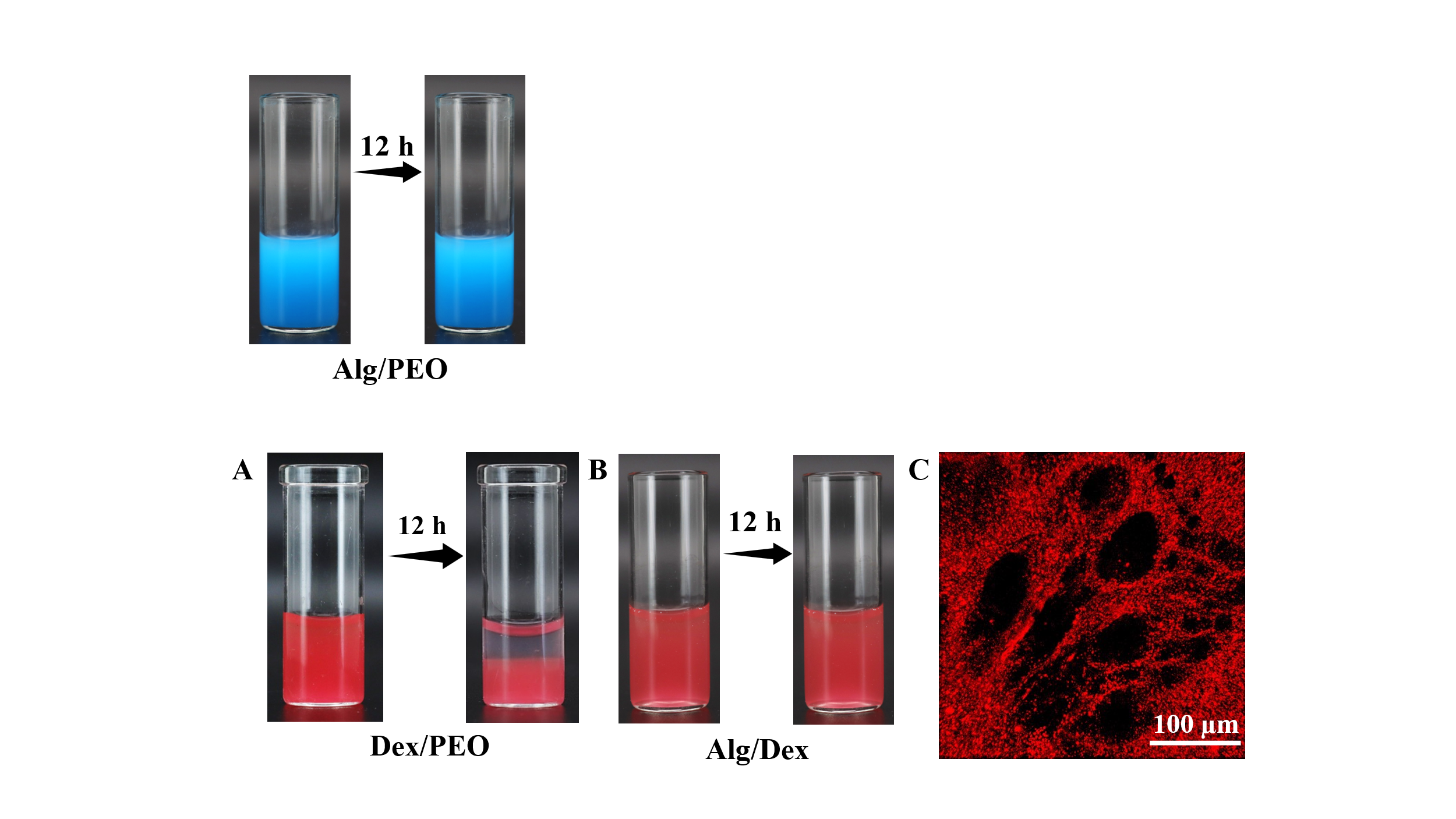


**Figure S1.** The exploration of aqueous two-phase systems. (A, B) Photographs of Dex/PEO and Alg/Dex phase separation after vortexing 12 hours standing; (C) Confocal fluorescence image of crosslinking Alg-Dex/PEO composite.


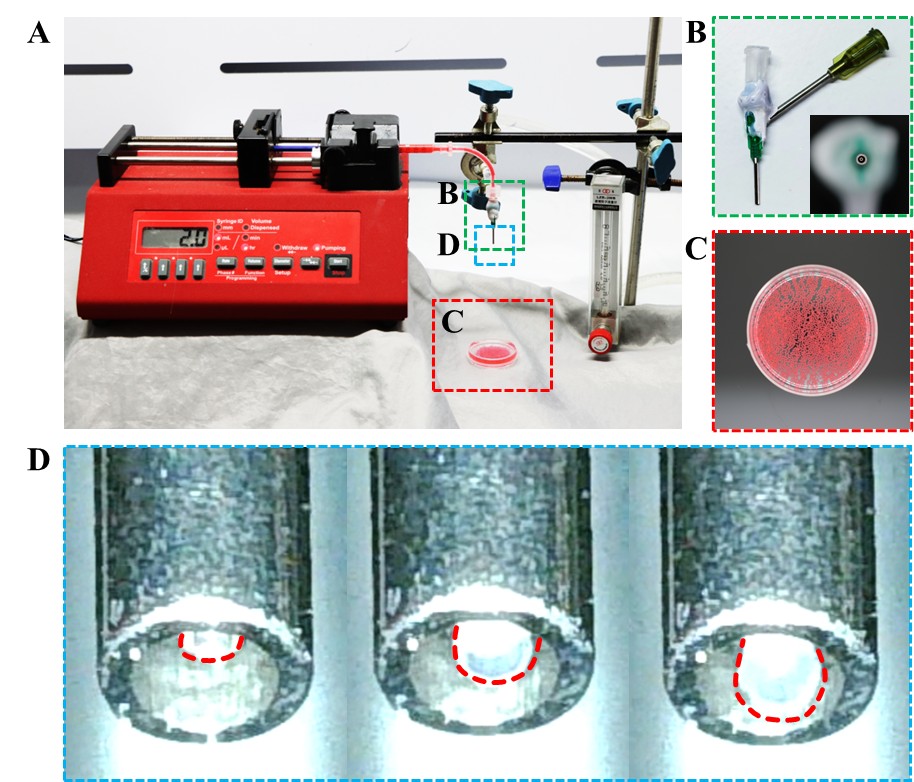


**Figure S2.** The gas-shearing microfluidic equipment and the microgels formation process. (A) Photograph of gas-shearing microfluidic equipment; (B) A coaxial needle; (C) A microgels collecting bath; (D) High-speed photography of the microgels formation process.


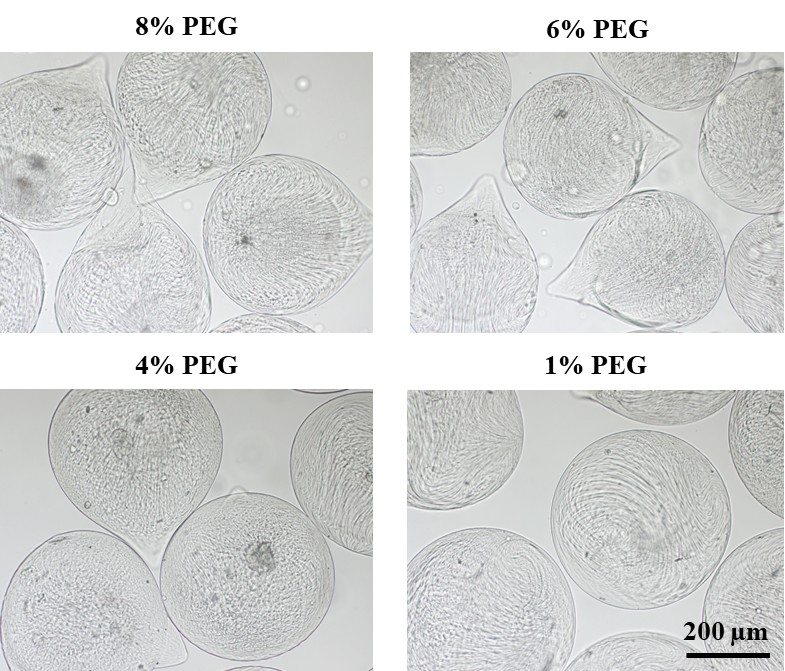


**Figure S3.** The photograph of porous microgels and the concentration of the collecting bath from 8% to 1% PEG with 2% Ca^2+^.


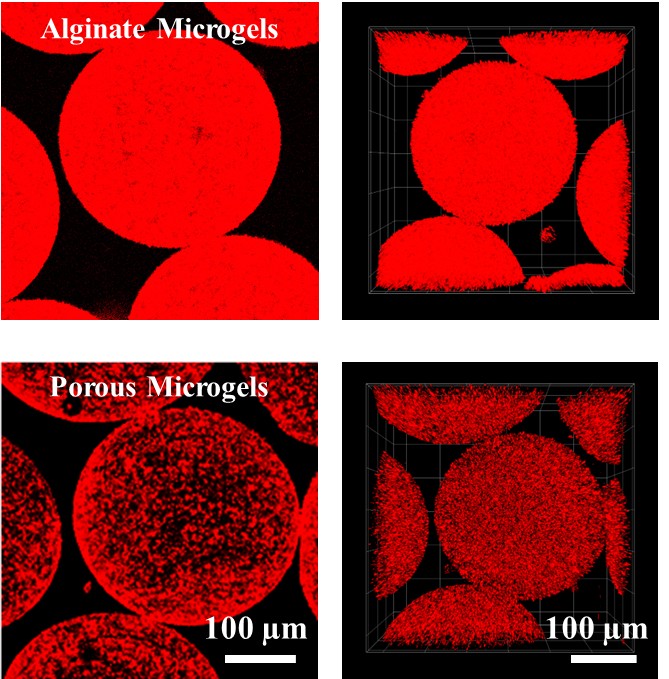


**Figure S4.** Fluorescence imaging of non-porous microgels and porous microgels.


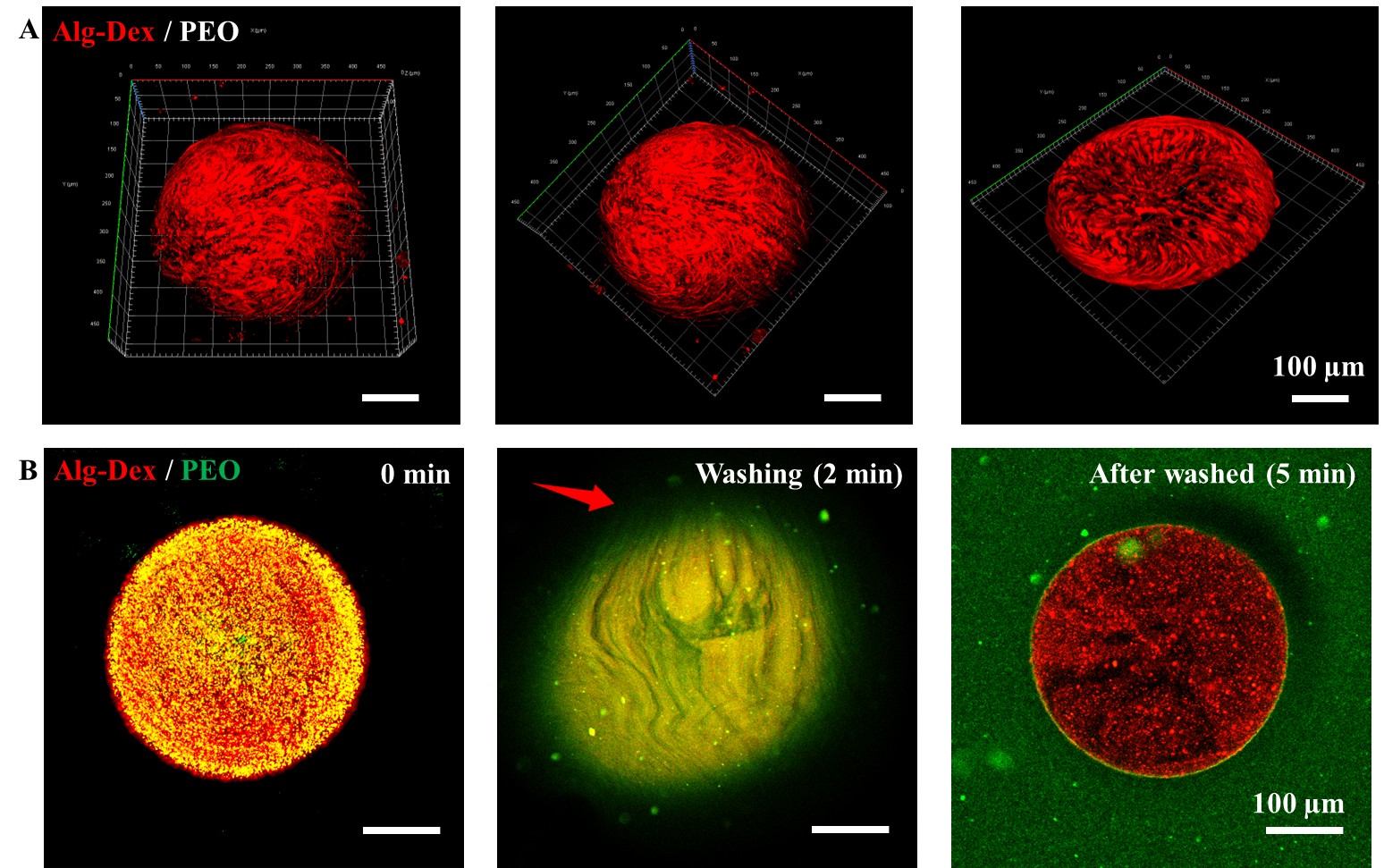


**Figure S5**. The microporous network structure of porous microgels. (A) Z-axis fluorescence scanning image of porous microgels; (B) Fluorescence image depicting the diffusion of PEO sacrificial phase within porous microgels and the red arrow indicates the dissolution of PEO.


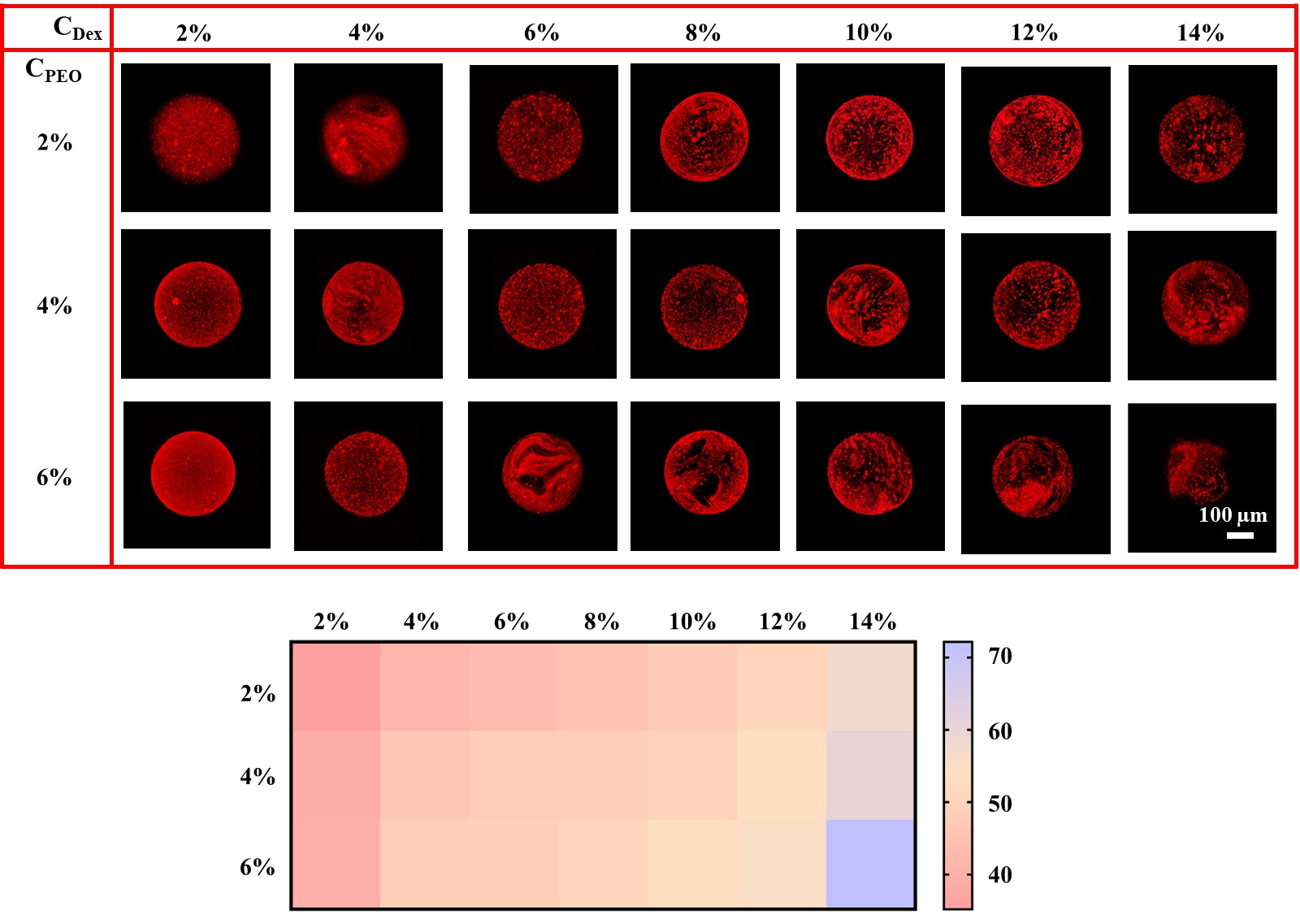


**Figure S6.** Porous microgels of different proportions by Dex and PEO.


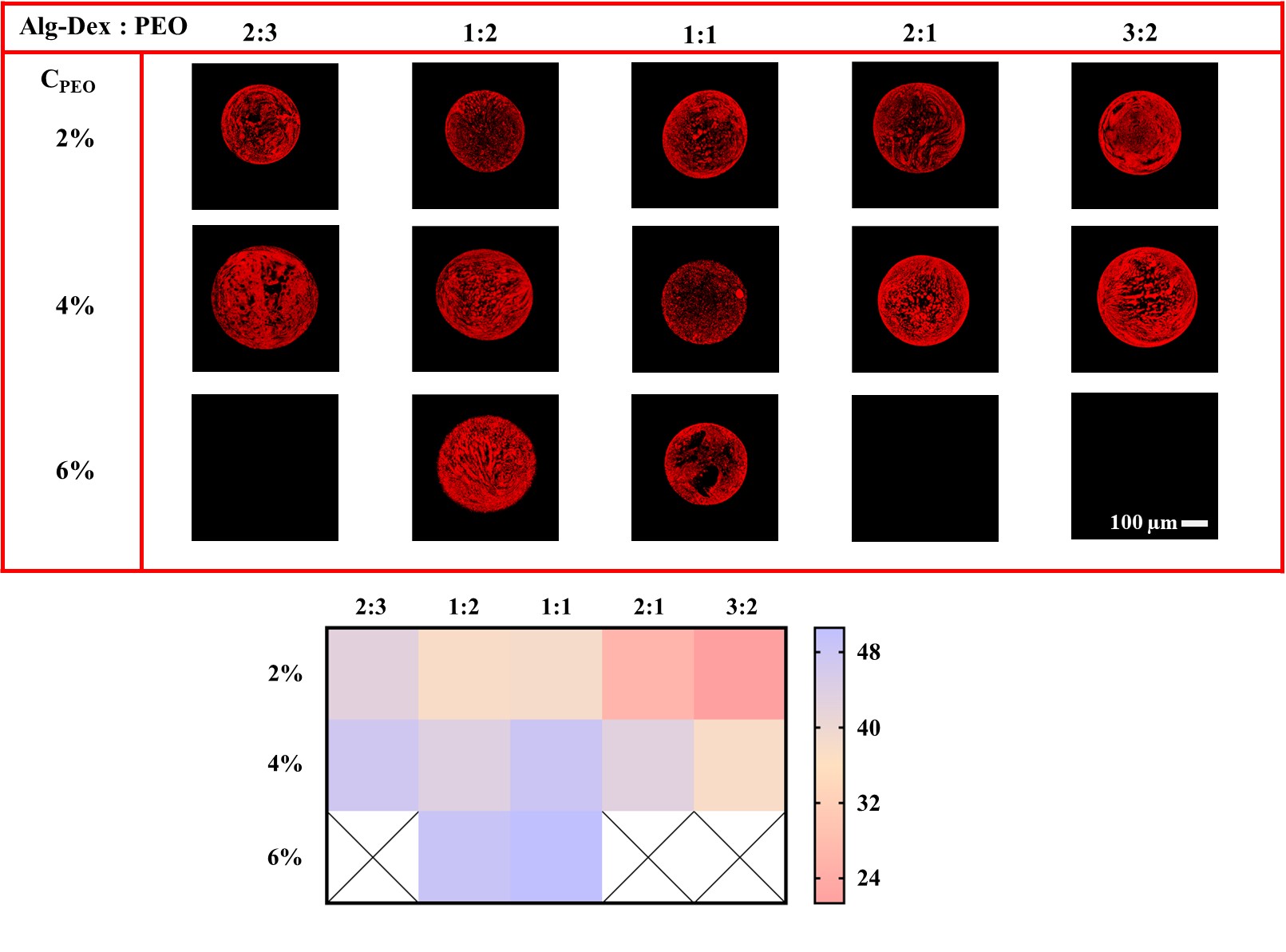


**Figure S7.** Porous microgels of different proportions by the rate of Alg-Dex and PEO.


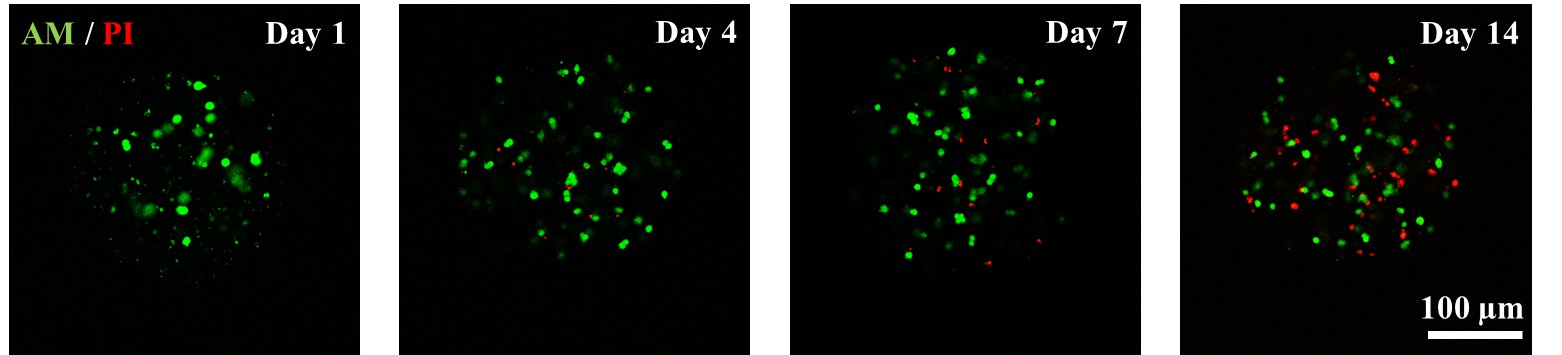


**Figure S8.** Fluorescence imaging of HUVECs-laden porous microgels over 14 days of culture by Live/Dead staining.


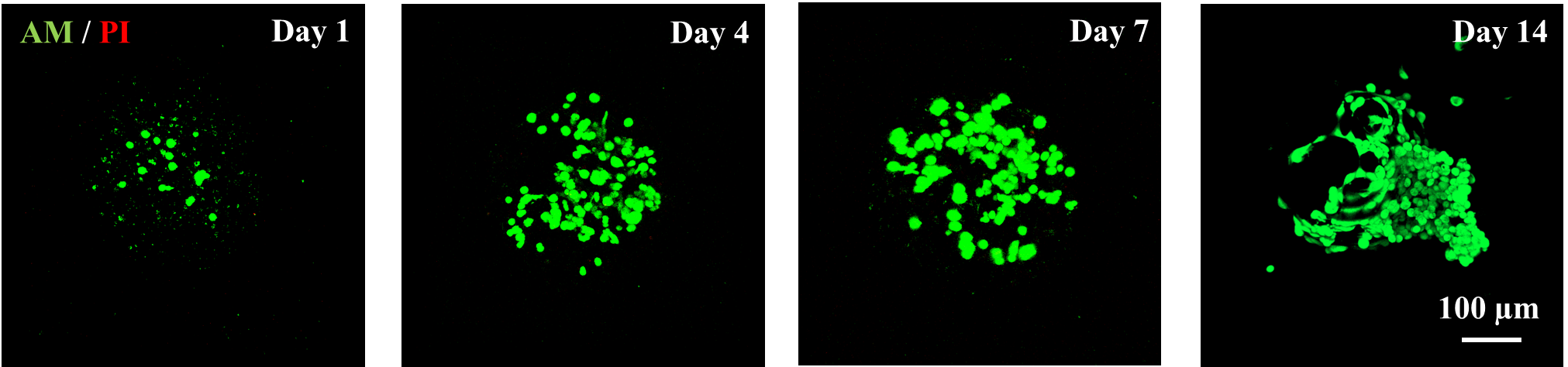


**Figure S9.** Fluorescence images of HUVECs-laden porous microgels over 14 days of culture by Live/Dead staining

.


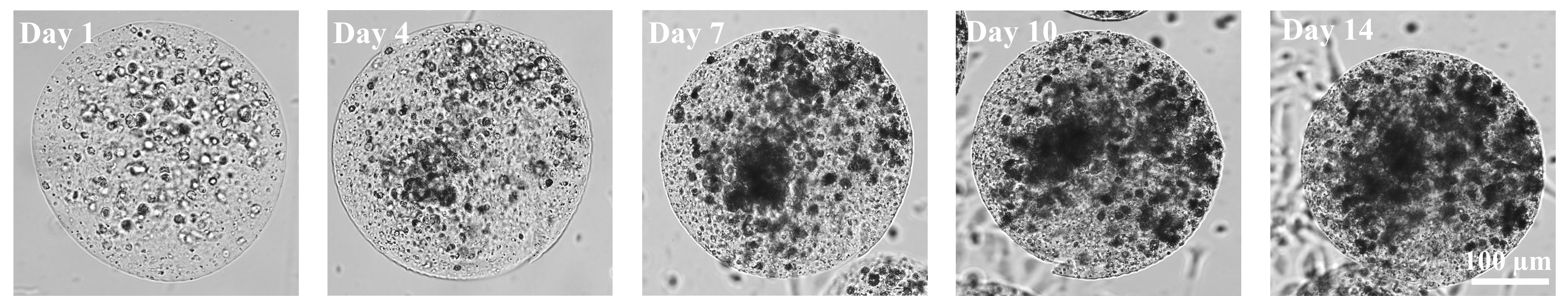


**Figure S10.** H9C2 culture in porous microgels over 14 days.


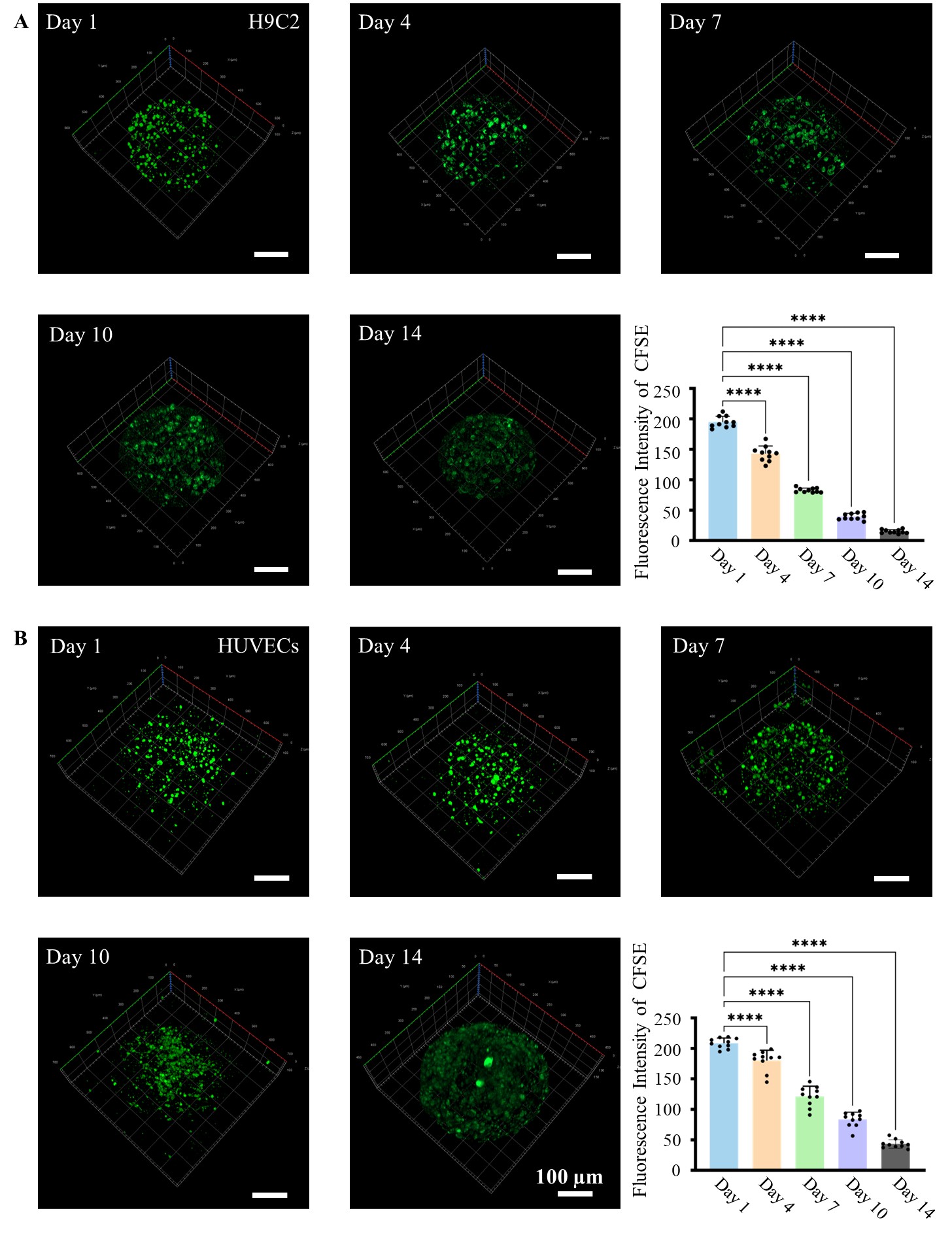


**Figure S11.** The proliferation of H9C2 (A) and HUVECs (B) in porous microgels over 14 days, n = 10, one-way ANOVA; *P < 0.05, **P < 0.01, ***P < 0.001, ****P < 0.0001. Data are presented as mean values ± SDs (compared with respective the Day 1).


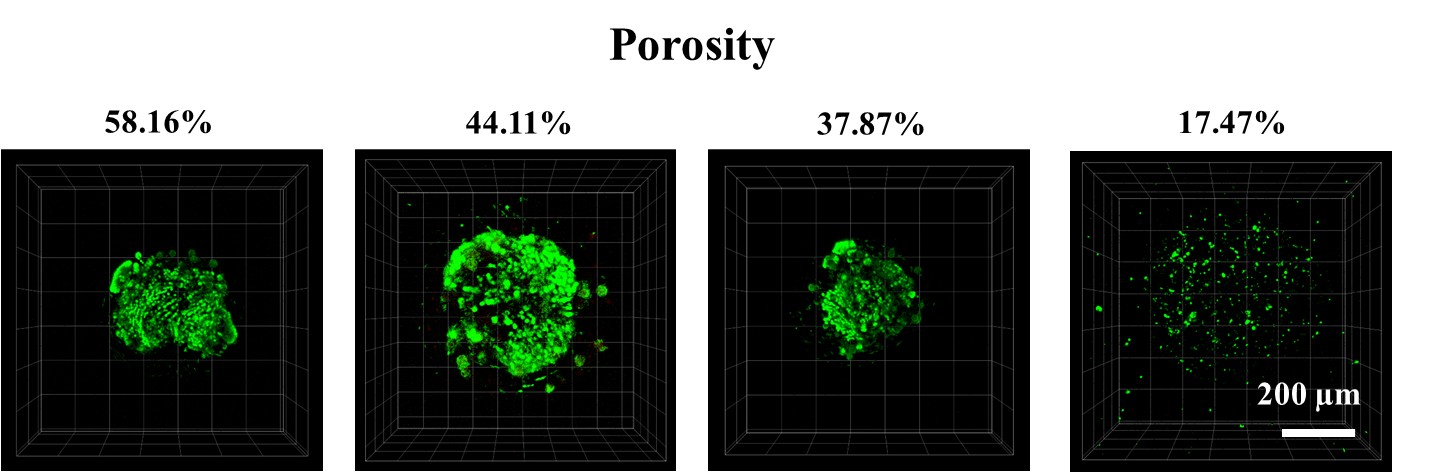


**Figure S12.** HUVECs cultured in porous microgels with different porosity by Live/Dead staining.


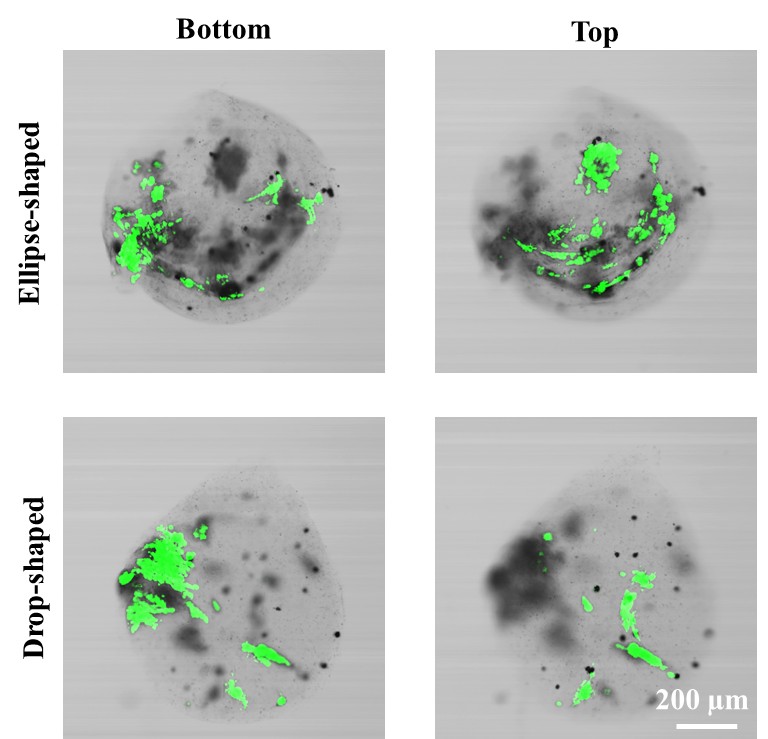


**Figure S13.** H9C2-laden porous microgels with two different morphologies (ellipse-shaped and drop-shaped) via 4% and 6% PEG collecting bath over 7 days of culture by Live/Dead staining.


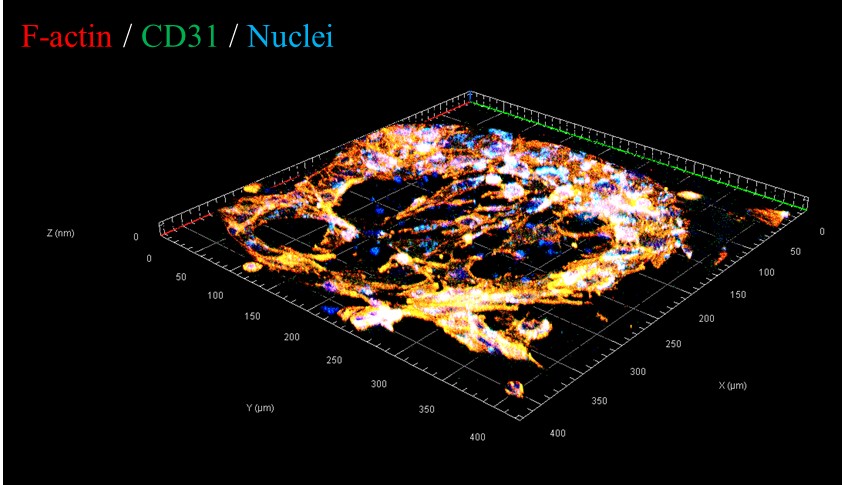


**Figure S14**. 3D Fluorescence images of the HUVECs-laden porous microgels at day 14 culture stained for F-actin (red), CD31 (green), and nuclei (blue).


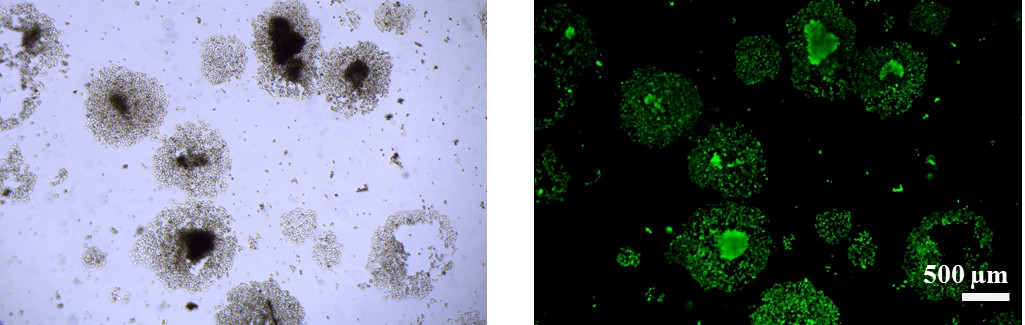


**Figure S15.** Photograph and fluorescence image of HeLa-laden porous microgels at 7 days of culture by Live/Dead staining.


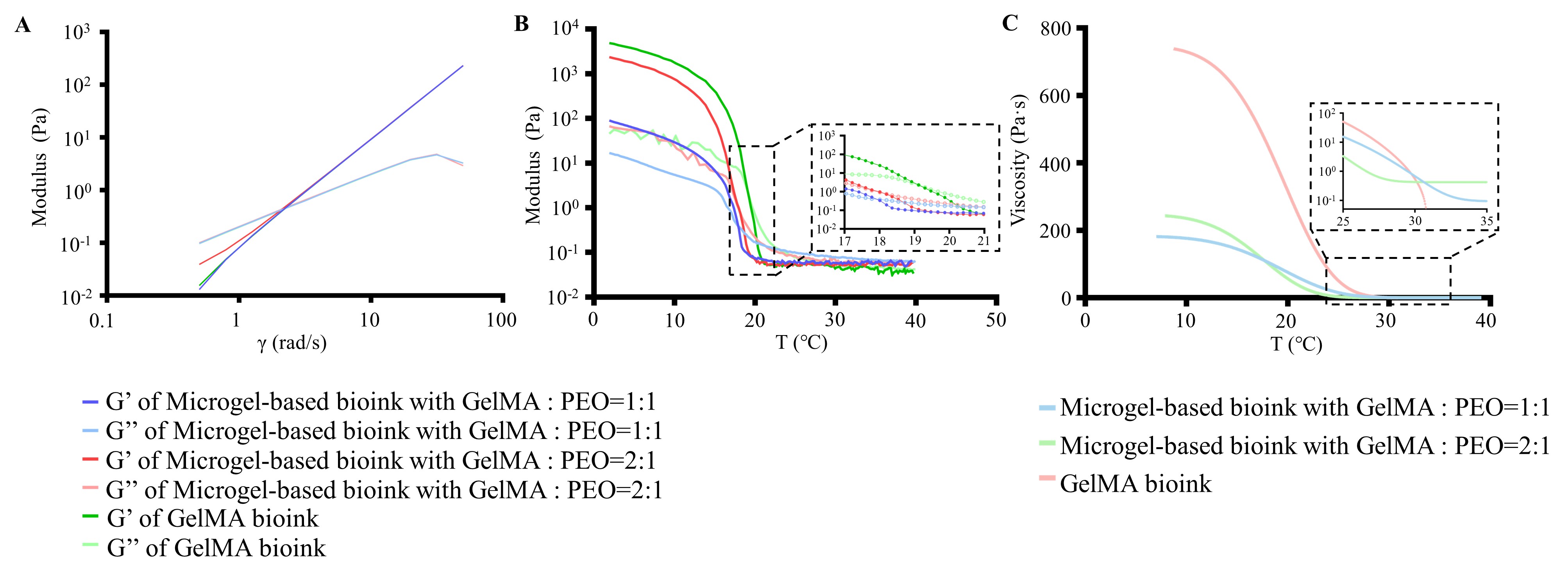


**Figure S16.** Rheological testing of microgel bioinks. (A) Shear frequency curve under 37 ℃; (B) temperature-modulus curve under 1 Hz; (C) Viscosity curve under 1 Hz.


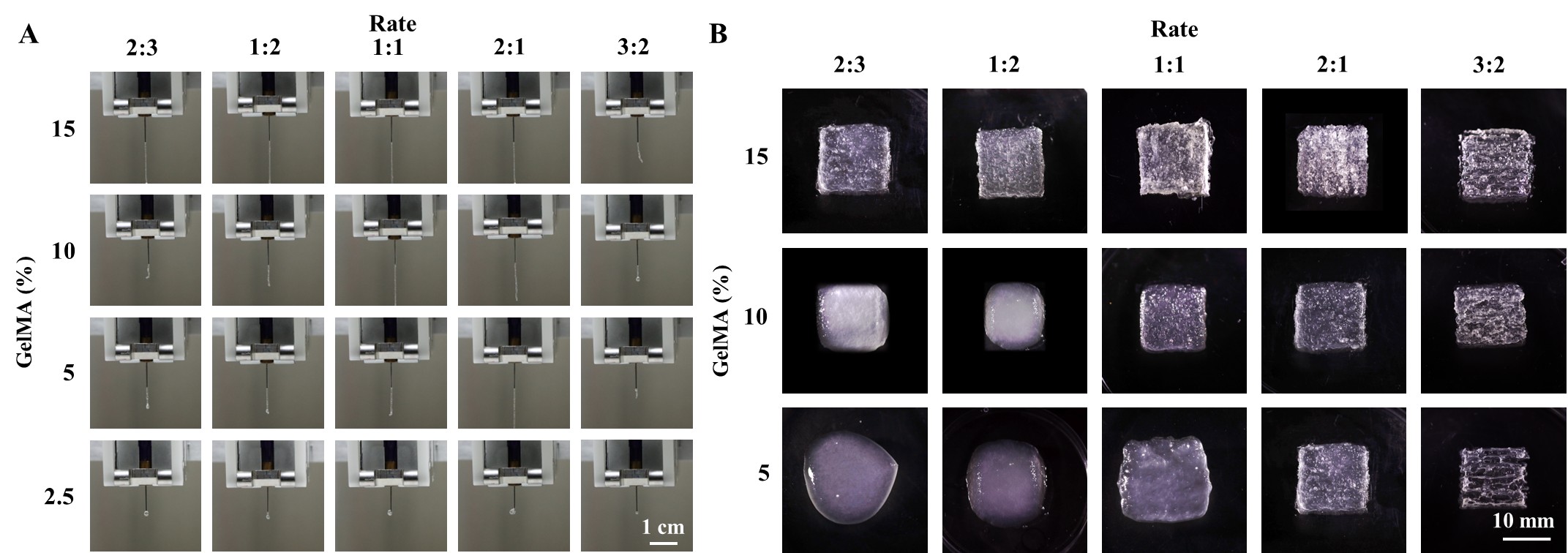


**Figure S17.** The extrusion printability of microgel-based bioinks.


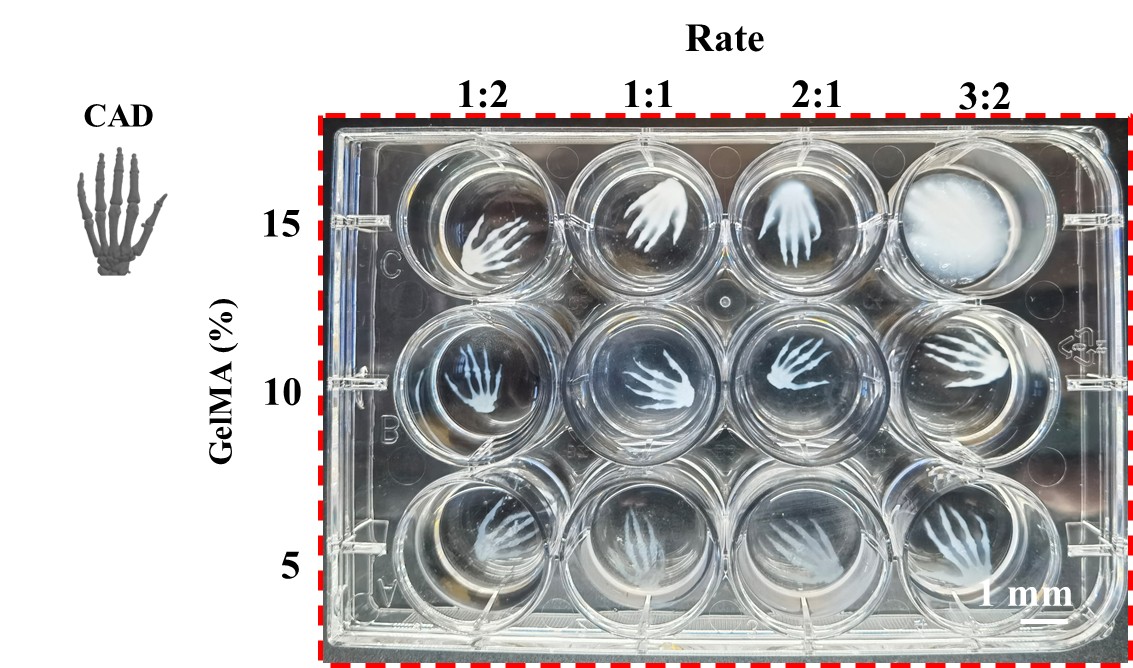


**Figure S18.** The DLP printability of microgel-based bioinks.


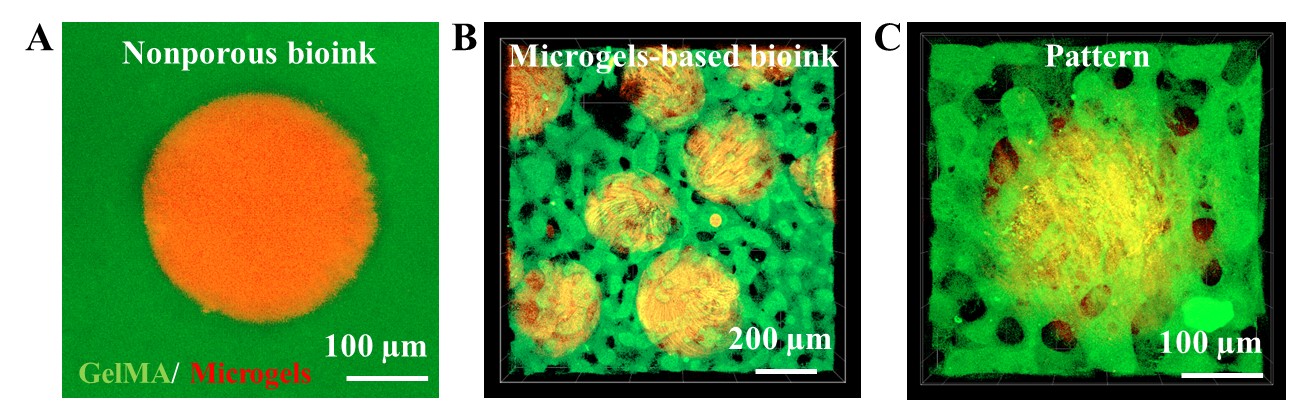


**Figure S19.** Fluorescence imaging of 3D printing. (A) Fluorescence image of nonporous bioink with pure GelMA and Alg microgels; (B, C) Fluorescence 3D image of hierarchical porous microgels-based bioink and printing pattern.


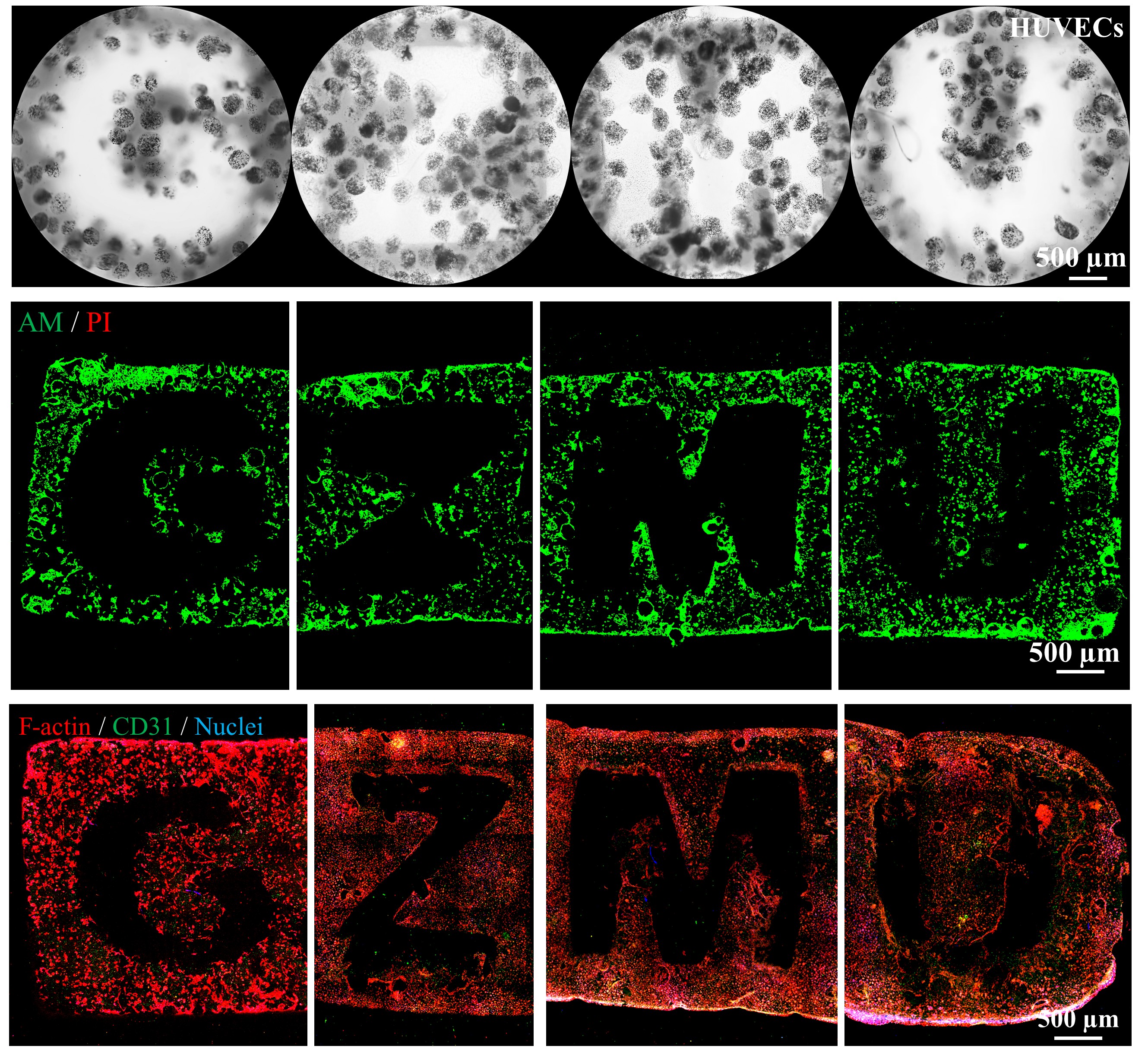


**Figure S20.** The cellular behavior of the printing constructs with HUVECs-loaded porous microgel-based bioink, via live/dead assays and immunofluorescence staining by F-actin (red) and CD31 (green) at day 7.


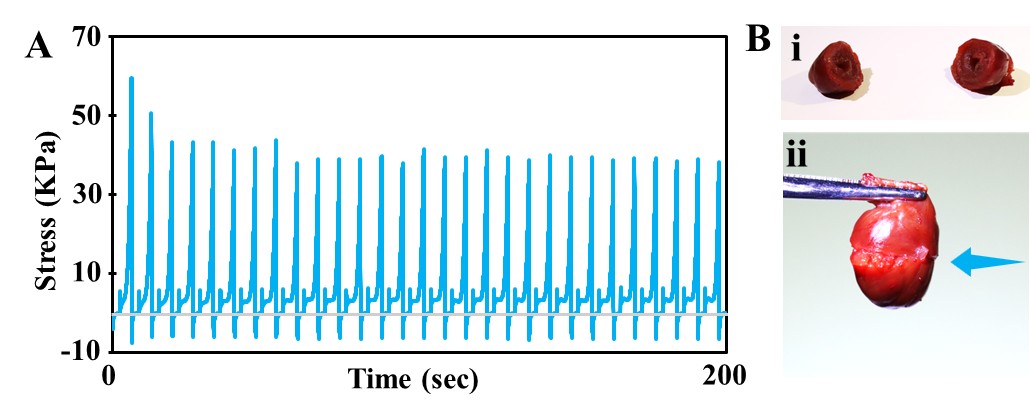


**Figure S21.** Adhesion of HPMP and pure GelMA patch. (A) Compression-separation stress time history curve; (B) The multi-level porous hydrogel patch adhered to the separated heart.


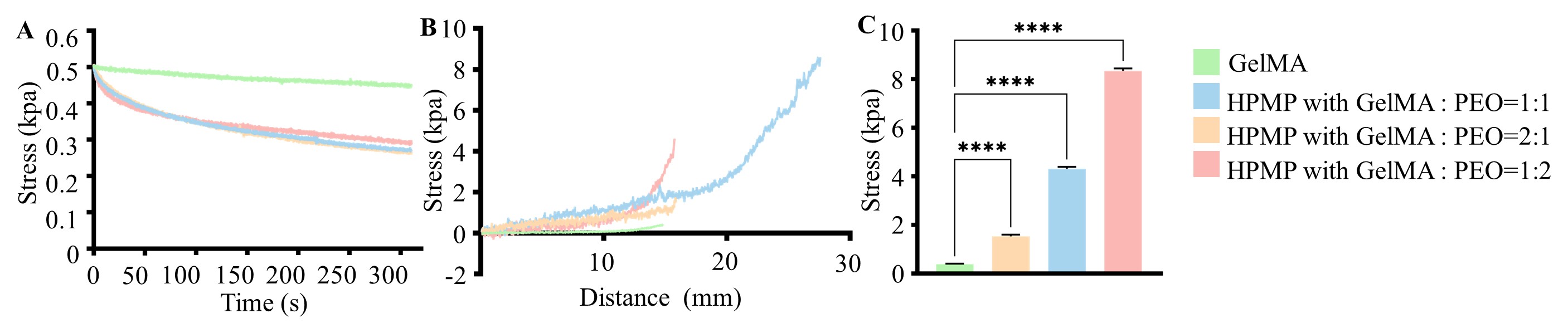


**Figure S22.** (A) Stress Relaxation Testing of HPMP and GelMA construction; (B) Shear tensile test of HPMP and GelMA construction; (C) Statistics chart of adhesion force, n = 5; one-way ANOVA; **P* < 0.05, ***P* < 0.01, ****P* < 0.001, *****P* < 0.0001. Data are presented as mean values ± SDs (compared with respective the GelMA group).


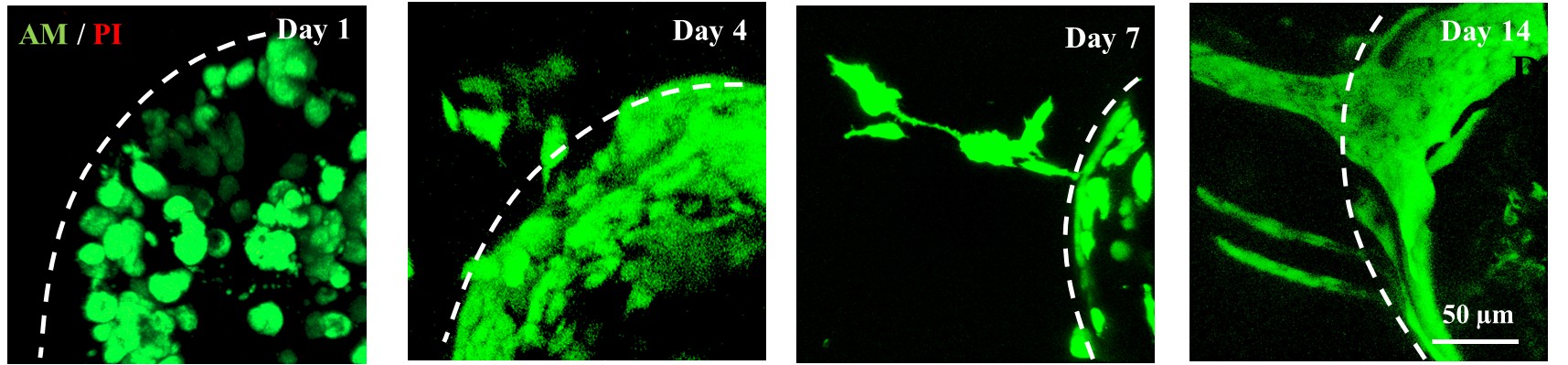


**Figure S23.** Fluorescence images of the HPMP with HUVECs-laden porous microgels of Live/Dead staining after 1, 4, 7, 14, 21 days of culture.


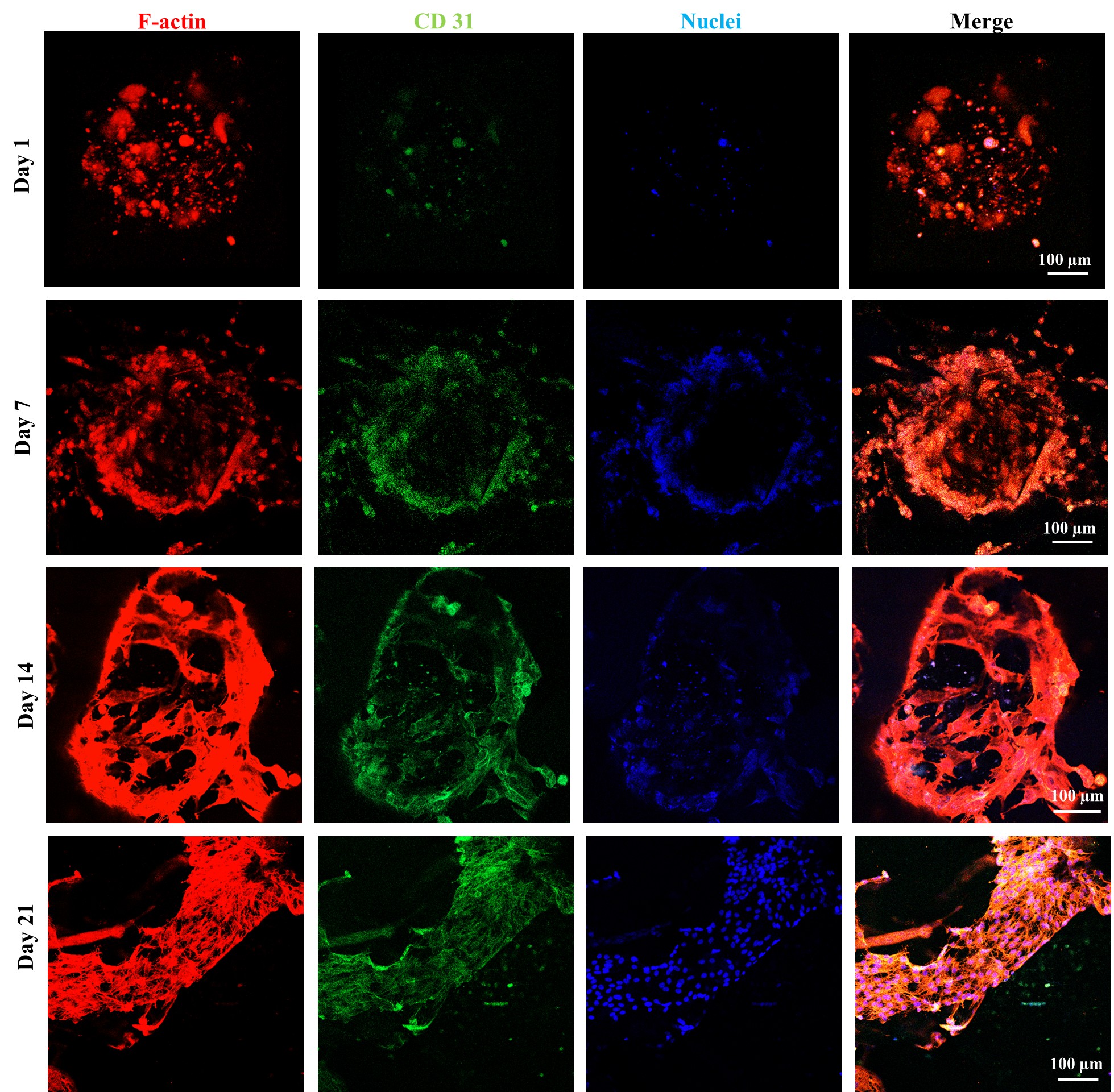


**Figure S24.** Fluorescence images of the HPMP with HUVECs-laden porous microgels after 1 7, 14, 21 days of culture, stained for F-actin (red), CD31 (green), and nuclei (blue).


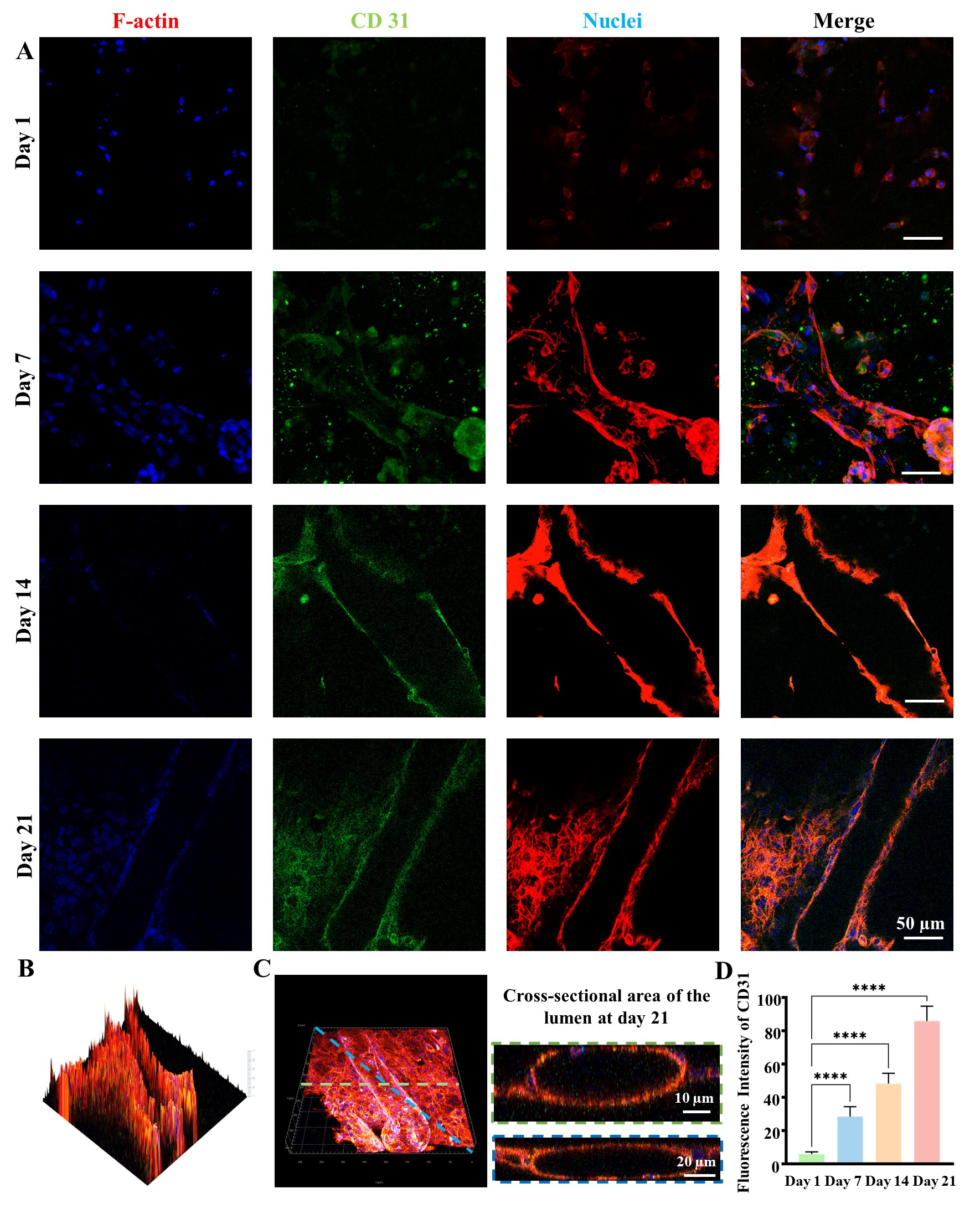


**Figure S25.** (A) Fluorescence images of the HPMP with HUVECs seeded on porous microgels over 21 days of culture, stained for F-actin (red), CD31 (green), and nuclei (blue). (B) The fluorescence intensity 2.5D graph and (C) 3D fluorescence image with cross-sectional views of lumens at day 21. (D) Statistical chart of quantitative fluorescence data for CD31, n = 5; one-way ANOVA; **P* < 0.05, ***P* < 0.01, ****P* < 0.001, *****P* < 0.0001. Data are presented as mean values ± SDs (compared with respective the Day 1).


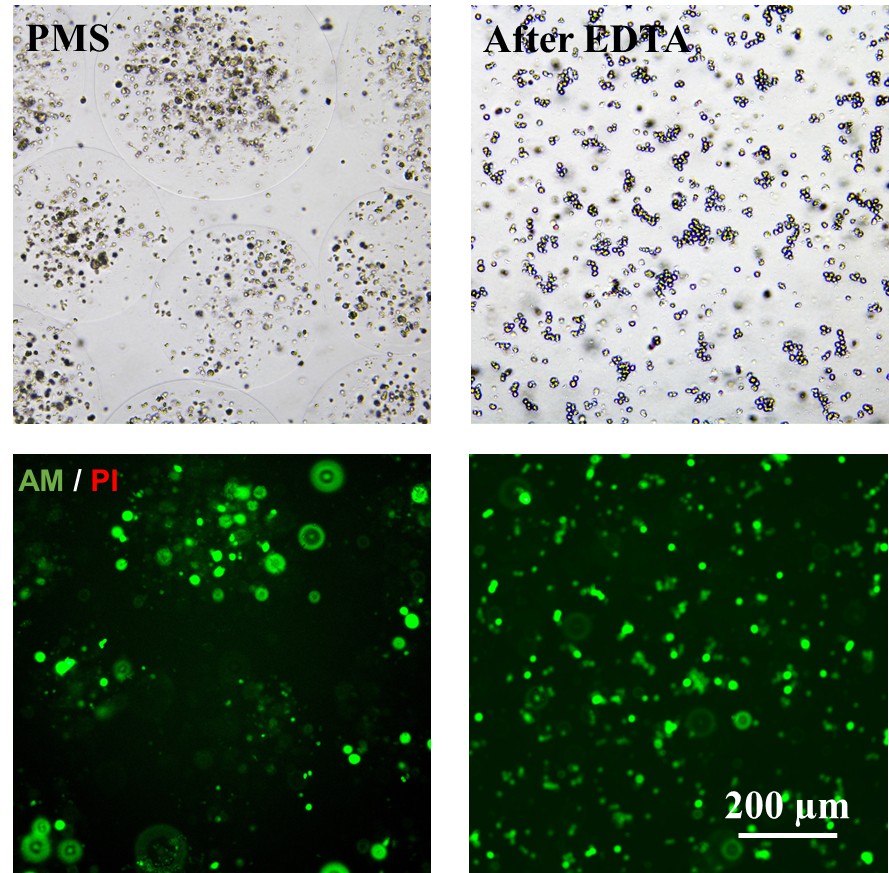


**Figure S26.** Fluorescence images of cell viability of iPSC-loaded porous microgels before and after EDTA treatment.


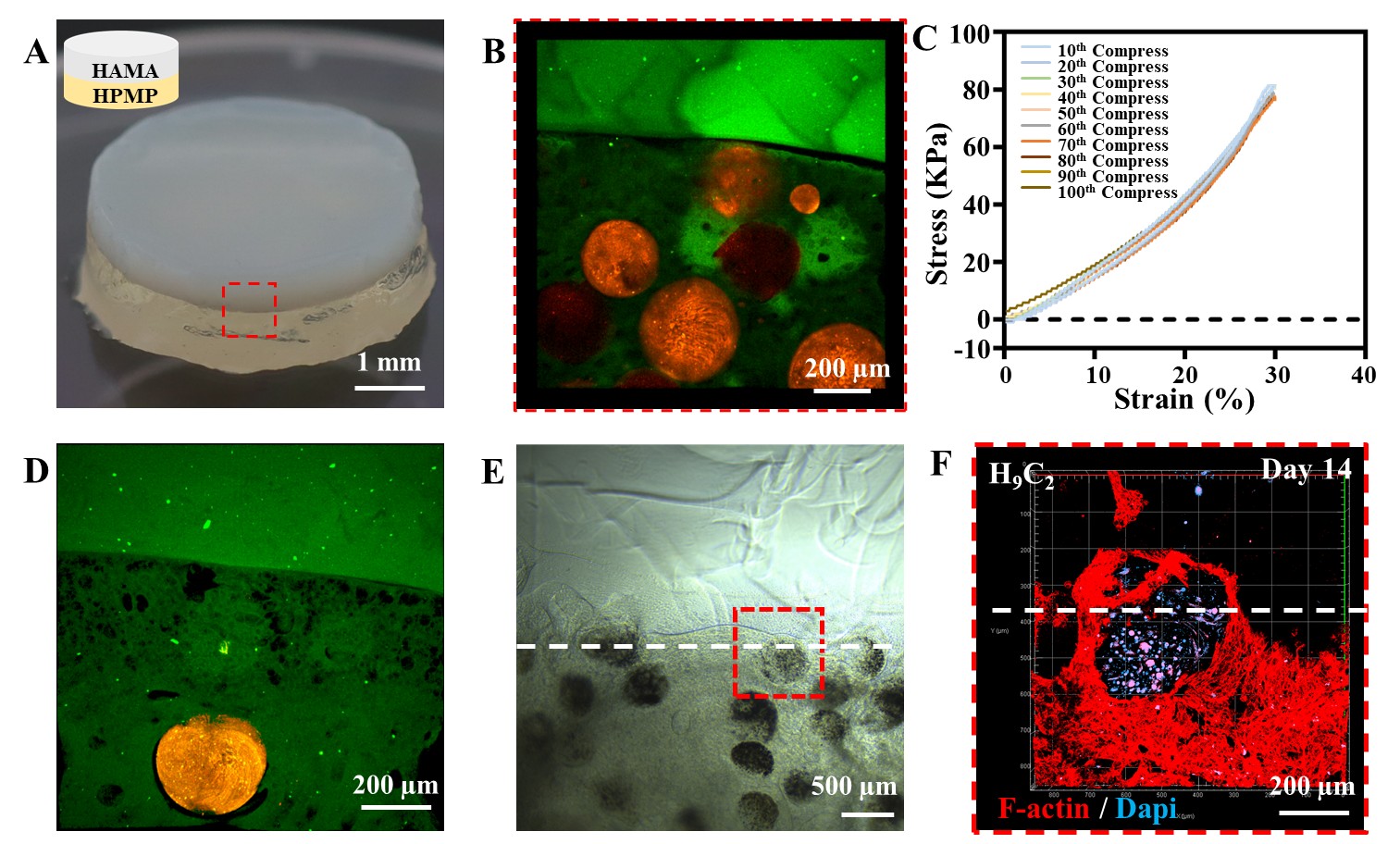


**Figure S27.** Fabrication and characterization of Janus porous microgels-based hydrogel (JHPMP). (A) Photograph of JHPMP; (B) Fluorescence images of the junction of JHPMP; (C) Stress-strain curve of porous microspheres under cyclic compression testing (testing conditions: strain of 50%, compression rate of 10 mm/s, 100 cycles); (D) Fluorescence images of the JHPMP after compression; (E, F) Photograph and fluorescence images of the JHPMP with H9C2-laden microgels over 14 days culture.


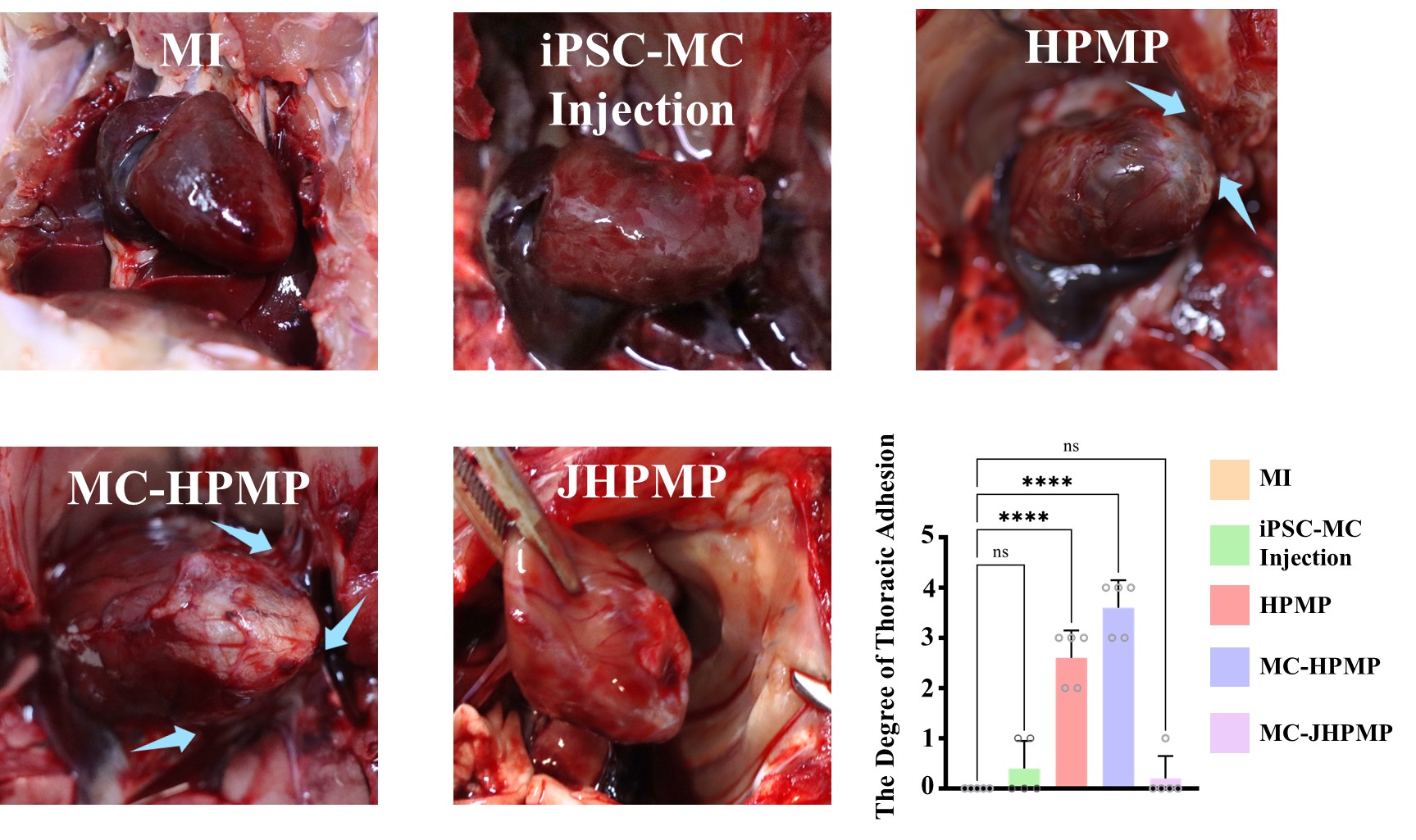


**Figure S28.** Photograph of heart after myocardial infarction treatment at day 28 for the adhesiveness of each group, and semi-quantitative analysis of the degree of thoracic adhesion, n = 5; one-way ANOVA; **P* < 0.05, ***P* < 0.01, ****P* < 0.001, *****P* < 0.0001. Data are presented as mean values ± SDs (compared with respective the MI group).


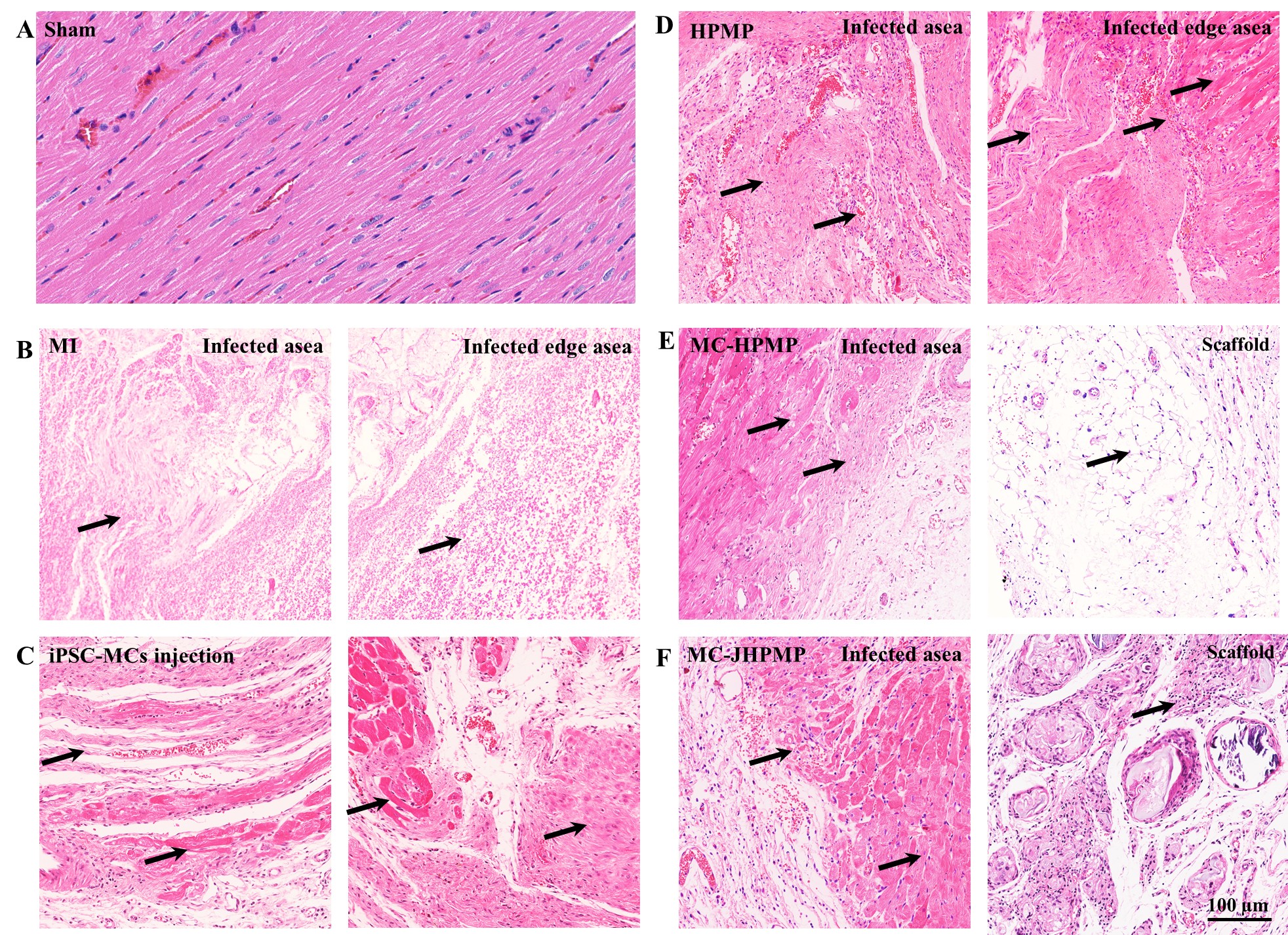


**Figure S29.** H&E staining at day 28 end point of sham group (A), MI group (B), iPSC-MCs injection group (C), HPMP group (D), MC-HPMP group (E), and MC-JHPMP group (F).


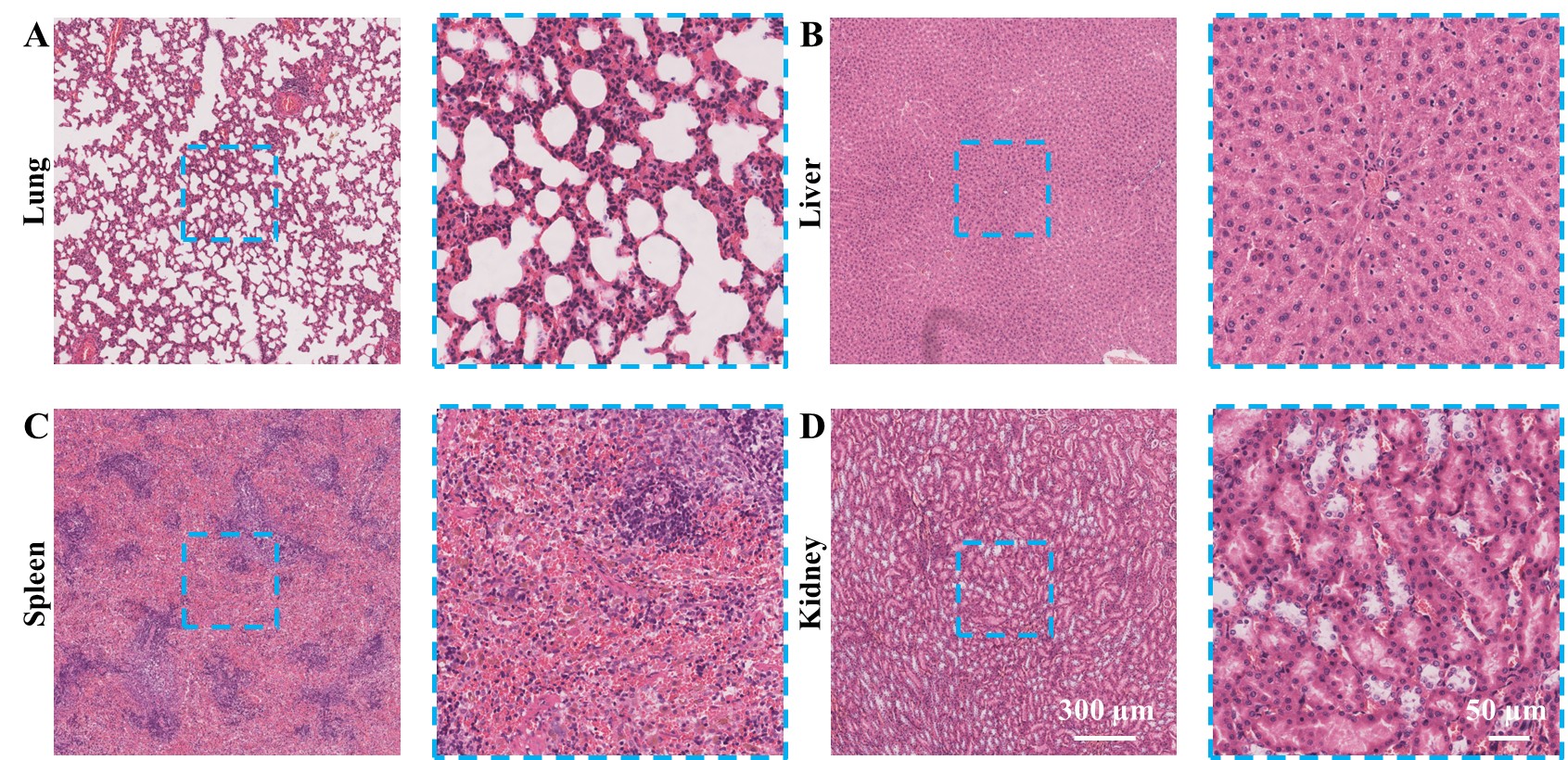


**Figure S30.** The biosafety of the transplanted HPMP via H&E staining of the lung (A), liver (B), spleen (C), and kidney (D).

**Table S1.** The primer sequences of RT-qPCR for cardiac differentiation.

| Gene | Forward primer (5’ - 3’) | Reverse primer (5’ – 3’) |
| --- | --- | --- |
| hsa-GAPDH | ACCACAGTCCATGCCATCAC | CATGCCAGTGAGCTTCCCGI |
| hsa-NKX2.5 | CTATCCGGGTTACGGCGG | TGAACCGCATTCAAGTCCCC |
| hsa-GATA4 | CGACACCCCAATCTCGATATGTT | ACAGATAGTGACCCGTCCCA |
| hsa-TBX5 | TACCACCACACCCATCAAC | ACACCAAGACAGGGACAGAC |
| hsa-MTH6 | CAAGAGCCGTGACATTGGTG | AGGTTGGCAAGAGTGAGGTT |
| hsa-cTnT | AGACGCCTCCAGGATCTGT | TCTTCAACAGCTGCTTCTTCC |

((Please insert your Supporting Information text/figures here. Please note: Supporting Display items, should be referred to as Figure S1, Equation S2, etc., in the main text…)
